# Arbuscular Mycorrhiza Induce the Regulation of Ca^2+^, ROS, and SOS Pathways Under Salt Stress in Tomato Roots

**DOI:** 10.1111/ppl.70610

**Published:** 2025-10-26

**Authors:** José Eduardo Marqués‐Gálvez, Luca Giovannini, Pierpaolo del Boccio, Fabiano Sillo, Elisa Zampieri, Chiara Pagliarani, Walter Chitarra, Silvia De Rose, Federico Vita, Raffaella Balestrini

**Affiliations:** ^1^ Institute of Bioscience and Bioresources National Council of Research Bari Italy; ^2^ Institute for Sustainable Plant Protection National Research Council (CNR‐IPSP) Torino Italy; ^3^ Research Centre for Viticulture and Enology Council for Agricultural Research and Economics (CREA‐VE) Conegliano Italy; ^4^ Department of Biology University of Bari ‘Aldo Moro’ Bari Italy

**Keywords:** antioxidants, ion homeostasis, mycorrhiza, Na^+^/K^+^ ratios, transcriptomics

## Abstract

Tomato ( *Solanum lycopersicum* L.) is a globally important horticultural crop, but its growth and productivity are limited by soil salinity. Arbuscular mycorrhizal fungi (AMF) are known to enhance salt tolerance in tomato, yet the underlying molecular mechanisms remain unclear. To explore this, we analysed the transcriptional response of non‐mycorrhizal and AMF‐inoculated tomato roots under salt stress. AMF‐inoculated roots showed altered regulation of genes involved in Ca^2+^ signalling and salt‐stress sensing, including *SlMOCA1* and components of the salt overly sensitive pathway such as *SlSOS3*, *SlTFT*, and *SlGI*. AMF also promoted ion homeostasis by altering the regulation of K^+^ transporters and *SlNHX* genes encoding vacuolar H^+^/Na^+^ exchangers, contributing to improved Na^+^/K^+^ ratios. Additionally, AMF enhanced the expression of genes involved in reactive oxygen species detoxification, including catalases and ascorbate peroxidases. These findings indicate that AMF inoculation supports salt‐stress tolerance in tomato roots through improved stress sensing, ion regulation, and antioxidant responses. This study provides new insights into the complex molecular interactions between tomato roots and AMF under salt stress, offering potential targets for breeding or gene‐editing strategies to improve crop resilience.

## 1 Introduction

The possibility for an individual to survive depends on their ability to respond adequately to environmental stresses. Plants, being sessile organisms, must develop strategies to resist unfavourable environments (Zhang et al. 2022). To activate adequate responses, plants perceive the incoming stress, communicate the alteration of the environment from the stressed tissue to the entire organism, and finally activate physiological responses (Zhu 2016; Zhang et al. 2020). Abiotic stressors, such as excessive heat, water deficit or excess, heavy metal deposits, and salt, have caused a reduction in the cultivable areas of agricultural crops in recent years, leading to a reduction in productivity (Yadav et al. 2019). Salinity is one of the most harmful abiotic stresses, causing reductions in terms of crop yield and quality (Atta et al. 2023). According to the FAO (2021), soil salinisation is becoming a greater problem in many areas, especially in arid and semi‐arid regions. Soils are classified as saline when their electrical conductivity (ECc) exceeds 4 dS m^−1^ (Munns and Tester 2008). Salinisation has been predicted to cause the loss of half of arable lands by the middle of the twenty‐first century (Mushtaq et al. 2020). Soil salinity initially causes osmotic stress due to a reduction in water potential in the soil, leading to cellular dehydration; subsequently, it results in ionic stress due to the accumulation of sodium in the cells, leading to ion toxicity, ultimately causing chlorosis and necrosis (Munns and Tester 2008; Acosta‐Motos et al. 2017). Plant salt‐stress responses at a molecular level include changes in transcriptional activity, alteration in protein levels, regulation of enzymatic activity, and production of secondary metabolites, as well as variations at the morpho‐functional level (Choi et al. 2014; Zhang et al. 2022).

The molecular mechanisms mediating plant salt‐stress responses include the activation of the salt overly sensitive (SOS) pathway. Briefly, under non‐stressful conditions, 14‐3‐3 (encoded by TFT genes) and GIGANTEA (GI) proteins negatively regulate the SOS pathway by repressing the protein kinase SOS2, which, in turn, activates the apoplastic Na^+^/H^+^ antiporter SOS1 (Yang and Guo 2018a, 2018b). However, salt stress is sensed through cell‐surface glycosyl inositol phosphorylceramide (GIPC) sphingolipids and triggers a cytosolic Ca^2+^ signal, finally resulting in the degradation of GI proteins and the activation of the EF‐hand Ca^2+^‐binding protein SOS3 and the aforementioned SOS2, activating SOS1 and the vacuolar Na^+^/H^+^ antiporters NHXs, which result in Na^+^ export out of the cytoplasm to avoid its toxic effects (Yang and Guo 2018a, 2018b; Jiang et al. 2019). Additionally, salt‐stress response also induces osmotic stress and production of reactive oxygen species (ROS). Plants are, however, able to counter this with the regulation of osmoprotectants, water transporters, that is, aquaporins, or antioxidant enzymes, such as peroxidases or catalases (Yang and Guo 2018a, 2018b).

Arbuscular mycorrhizal fungi (AMF), obligate biotrophs currently assigned to the phylum Glomeromycota (Wijayawardene et al. 2024), can form mutualistic symbioses with the roots of about 80% of terrestrial plant species, playing a key role in essential mineral nutrient acquisition and translocation, and receiving carbon compounds in return (Smith and Read 2010). AMF naturally occur in saline soils (Aliasgharzadeh et al. 2001), and even if salinity negatively impacts the mycorrhizal symbiosis formation and function (Juniper and Abbott 1993), it has been demonstrated that inoculation with AMF can positively influence the resilience of plants to salt (Sannazzaro et al. 2006; Jahromi et al. 2008; Estrada et al. 2013; Chandrasekaran et al. 2021; Cheng et al. 2021). AMF, in fact, can improve nutrient and water uptake, ion homeostasis, osmoregulation, stimulate the activity of antioxidant defence systems and endogenous hormone regulation, and changes in the rhizosphere microbial community (Ruiz‐Lozano and Azcón 1995; Asghari et al. 2005; Evelin et al. 2019; Bastías et al. 2022). Tomato ( *Solanum lycopersicum* L.) is an important horticultural crop, cultivated worldwide, used as food and feed ingredients, rich in vitamins A and C and carotenoids such as lycopene (Canene‐Adams et al. 2005; Cortina and Culiáñez‐Macià 2005). Although tomato is generally considered a moderately salt‐tolerant crop, with genotype‐dependent variation (Maas and Hoffman 1977; Dasgan et al. 2002) that goes from little tolerance, up to 100 mM of NaCl (Ali et al. 2021), salinity remains a major limitation to its cultivation (Yurtseven et al. 2005). It causes disruption of several physiological processes, reducing yield and plant growth, thus affecting plant length, fresh and dry shoot and root tomato weight (He et al. 2007; Tanveer et al. 2019). AMF have previously been shown to act at different levels contributing to salt‐stress tolerance in tomato. For instance, the AM symbiosis in two different tomato cultivars (*cv*. Behta and *cv*. Piazar) enhanced growth compared to not‐inoculated plants, due to reduced Na^+^ accumulation in the roots and increased antioxidant enzyme activity; the effect of AM fungal inoculation appeared to be genotype‐dependent (Hajiboland et al. 2010). Salt‐stressed (150 mM NaCl) tomato (*cv* Huapiqiu), colonised by *Paraglomus occultum*, showed better tolerance than the non‐inoculated ones. Fungal symbiosis altered the expression levels of cytoplasmic (PIPs) and tonoplastic (TIPs) aquaporins and reduced the expression levels of *SOS1* and *SOS2* genes compared to non‐inoculated plants (Liu et al. 2023).

In a recent work, we reported that *Funneliformis mosseae* inoculation modifies tomato (*cv*. Moneymaker) physiological response to salinity at the shoot level. Leaf gas‐exchange parameters in response to salt stress were enhanced in *F. mosseae*‐inoculated plants. Moreover, gene expression and metabolite abundance profiles assessed in the leaves pointed to a substantial reprogramming of the primary metabolism, including aromatic amino acids and osmoprotectants (Giovannini et al. 2024). However, the molecular mechanisms underlying the enhanced salt‐stress tolerance at the root level in AMF‐inoculated plants were not assessed. Therefore, the present study aims to evaluate the effect of inoculation with *F. mosseae* on tomato (*cv*. Moneymaker) roots under salt stress. Here, combined molecular approaches (RT‐qPCR and RNA‐seq) and ion concentration analyses have been applied to test whether the AMF‐related benefits on salt tolerance are associated with differences in salt‐stress sensing, signalling and response in tomato roots. This work mainly focuses on evaluating how AMF inoculation impacts the regulation of salt‐stress sensing, ionic stress and ROS detoxification pathways, aiming to improve our overall understanding of the processes of AM‐induced salt‐stress tolerance in tomato plants.

## 2 Materials and Methods

### 2.1 Plant Material and Experimental Set‐Up

Tomato roots (*cv*. Moneymaker provided by Semiorto Sementi S.r.l.) grown in the presence (MYC) and in the absence (NMYC) of the AM fungus (AMF) were obtained from a previous experiment (Giovannini et al. 2024). Briefly, seeds were germinated in 0.7 L pots filled with pumice at the bottom, followed by coconut fibres and a ring with sterilised (at 180°C for 3 h) quartz sand and coconut fibres at the top (not inoculated, NMYC). For AMF (MYC) pots, the inoculum was mixed with the sterile sand ring, using 100 mL of inoculum with 300 mL of sterile sand (1:4) for four pots. The pots were watered two times a week with tap water and once a week with ½ Hoagland solution (Hoagland and Broyer 1936), starting 1 month after sowing. Plants were cultivated in a glasshouse, with mean daily temperatures of 24.9°C ± 5.4°C, relative humidity of 42.3%–61.8% and maximal photosynthetic photon flux density (PPFD) of 900–1200 μmol photons m^−2^ s^−1^. When necessary, a photoperiod of 12 h light–12 h dark was obtained with halogen lamps to guarantee a minimum PPFD of 500–600 μmol photons m^−2^ s^−1^. One and a half month after sowing, NMYC and MYC plants were subjected to salt stress (SS) conditions by means of increasing concentrations of NaCl solution, providing 30 mL for each pot. The NaCl concentration was gradually increased every 3 days first to 50 mM, until reaching the maximum concentration (200 mM), according to Pollastri et al. (2018). The maximum concentration was maintained for 11 days, administered every 3 days. After 20 days of stress, physiological parameters were measured, and root samples were collected for molecular analyses. Not‐stressed (NS) and not‐inoculated (NMYC) plants were considered as controls. In total, four treatments, including Non‐Mycorrhizal‐Not‐stressed (NMYC_NS), Mycorrhizal‐Not‐stressed (MYC_NS), Non‐Mycorrhizal‐Salt‐stressed (NMYC_SS) and Mycorrhizal‐Salt‐stressed (MYC_SS), were evaluated. Root samples were quickly collected, flash‐frozen in liquid nitrogen and stored at −80°C until they were processed. More than 2 months after seedling inoculation, mycorrhizal colonisation was checked on three plants under SS and NS conditions. Roots were stained with 0.1% cotton blue in lactic acid, and the fungal colonisation was verified using the method described by Trouvelot et al. (1986).

### 2.2 Biometric, Gas‐Exchange and Metabolic Parameters

Biometric (plant height, number of leaves and basal stem diameter), gas‐exchange rates (stomatal conductance ‐*g*_s_‐, transpiration ‐E‐, and net carbon assimilation ‐A_N_‐), chlorophyll content index (CCI) and metabolic (salicylic acid—SA, jasmonic acid ‐JA, abscisic acid—ABA, indole acetic acid—IAA, and proline leaf content) parameters were measured from NMYC_NS, NMYC_SS, MYC_NS and MYC_SS plants at the end of the salt stress treatment, as explained in Giovannini et al. (2024). These 12 parameters were used to perform a principal component analysis (PCA) to inspect the overall effect of AM inoculation on plant stress responses. Data related to stress‐related metabolites, including phenylalanine, arginine, pyroglutamic acid, prodelphinidin A1, hydroxycoumarin, 1‐caffeoylquinic acid, ascorbic acid, t‐aconitate, quinic acid, fructose, and OPDA containing galactolipid, were extracted from Giovannini et al. (2024).

### 2.3 Quantitative Gene Expression Analysis of Roots

Expression changes of the target transcripts were quantified in root samples by RT‐qPCR. Three biological replicates for each condition (NS and SS), inoculated (MYC) and not‐inoculated (NMYC) plants, were considered for the experiment. Total RNA for each sample was used to synthesise the cDNA, according to the SuperScript II Reverse Transcriptase (Invitrogen) procedure using random primers. RNA samples were previously treated with the TURBO DNase kit (Thermo Fisher Scientific), and genomic DNA contamination was checked before proceeding with cDNA synthesis by one‐step RT‐PCR using *SlCAC‐specific* primers of tomato (Table S1). Reactions were carried out in the ConnectTM Real‐Time PCR Detection System (Bio‐Rad Laboratories), and the SYBR Green method (Power SYBR Green PCR Master Mix; Bio‐Rad) was used to quantify the amplification results. Thermal cycling conditions were as follows: an initial denaturation phase at 95°C for 10 min, followed by 40 cycles at 95°C for 15 s and 60°C for 1 min. Specific annealing of primers was checked using dissociation kinetics performed at the end of each RT‐qPCR run. Gene expression data were calculated as expression ratios (relative quantity) using control data from uninoculated plants. The expression of tomato target transcripts was quantified after normalisation to two established reference genes in this species (*SlCAC* and *SlExpressed*; González‐Aguilera et al. 2016). These genes have been identified as stable reference genes across different tissues and development stages (Expósito‐Rodríguez et al. 2008; Wang et al. 2018), showing a standard deviation of ~10% from their average expression, and were not found among the differentially expressed genes in our RNA‐seq dataset (Table S2), thus confirming their stability within our experimental setup. The selected genes for gene expression studies were: *SlSOS1* encoding a Na^+^/H^+^ antiporter (Liu et al. 2023), *SlSOS2* encoding a serine/threonine protein kinase (Liu et al. 2023), a gene family including *SlRBOH1*, *SlRBOHD*, *SlRBOHF* encoding a NADPH membrane oxidase (Raziq et al. 2022), a gene family encompassing *SlNHX1*, *SlNHX2*, *SlNHX3*, *SlNHX4* encoding a vacuolar Na^+^/H^+^ antiporter (Gálvez et al. 2012), *SlHKT1.2* encoding a sodium transport (Jaime‐Pérez et al. 2017), *SlPOD* encoding a peroxidase enzyme (Raziq et al. 2022), *SlPIP1;2*, *SlPIP1;5*, *SlTIP2;2*, *SlTIP3;2* belonging to the aquaporin family (Liu et al. 2023), *SlCAT1* encoding a catalase enzyme (Pascual et al. 2023), *SlFeSOD1* encoding an iron superoxide dismutase enzyme, which plays a pivotal role in mitigating oxidative stress by scavenging superoxide radicals (Pascual et al. 2023), *SlCuZnSOD1* and *SlCuZnSOD2* encoding antioxidant enzymes involved in the detoxification of superoxide ions ($O_{2}^{-}$; Pascual et al. 2023). Gene‐specific primers are listed in Table S1.

### 2.4 RNA Extraction, Library Preparation and Sequencing

Total RNA was extracted from 0.1 g of root material according to the CTAB‐based method (Chang et al. 1993). For RNA‐seq analysis, three conditions (three biological replicates each) were considered: NMYC_NS (control condition), NMYC_SS (to assess the salt‐stress response in not‐inoculated plants), and MYC_SS (to assess the AMF‐mediated salt stress response in inoculated plants). The quantity and quality of the extracted RNA samples were determined using a Nanodrop 2000 spectrophotometer (Thermo Fisher Scientific). RNA integrity was assessed on an RNA 6000 Nano Labchip using a Bioanalyzer 1000 (Agilent Technologies) prior to library preparation. Only samples showing an RIN (RNA Integrity Number) value > 8 were processed for sequencing. Library preparation and RNA sequencing were performed at an external service (IGATech s.r.l laboratories). In detail, the Universal Plus mRNA‐Seq kit (Tecan Genomics) was used for library preparation following the manufacturer's instructions and sequenced on an Illumina NovaSeq 6000 apparatus (Illumina). Trimming of lower‐quality bases and adapters was performed using the ERNE software according to del Fabbro et al. (2013).

### 2.5 RNA‐Seq Data Analysis

For alignment, reads were mapped onto the reference genome GCF_000188115.5_SL3.1 (Hosmani et al. 2019) using STAR v. 2.7.10 (Dobin et al. 2013). The software htseq‐count v. 2.0.2 (Anders et al. 2015) was utilised to count the overlapping of reads with genes. The data were then used to identify differentially expressed genes (DEGs) using the DESeq2 package v1.34.0 (Love et al. 2014) in R. The variance on read count was calculated based on three biological replicates *per* condition, by applying a negative binomial distribution to model the count data, therefore identifying genes showing significant expression changes among the different tested conditions. The DEG identification was performed after normalisation of the count data and correction for multiple testing, both accounted for by DESeq2, through the Wald test. During DESeq2 analysis, the shrinkage estimation of effect size (LFC estimates) was used to generate more accurate Log2 fold change (Log2FC) estimates, considering the variability among replicates. A cut‐off of the *p‐*adjusted value < 0.05 was used to classify a gene differentially expressed (DEG) in comparison with the reference (i.e., non‐mycorrhizal‐not‐stressed, NMYC_NS). Both the identified DEGs and all transcripts of the tomato transcriptome were annotated through Blast2GO v5.2.5 (Conesa et al. 2005) to obtain an updated functional annotation and to assign the corresponding Gene Ontology (GO) terms. A gene class functional enrichment analysis was then conducted using Blast2GO to reveal the biological processes, pathways, or other functional categories that are enriched among the identified DEGs. In addition, Kyoto Encyclopedia of Genes and Genomes (KEGG) enrichment analysis of gene clusters was performed by using the ClusterProfiler package v4.12.6 (Yu et al. 2012) in R, with *p* value and *q* value cutoffs ≤ 0.05 and 0.2, respectively.

### 2.6 Analysis of Ion Concentration in Stems

Stems of SS plants were dried at 50°C for 1 week, to assess the effect of AMF on NaCl accumulation in the stem. The concentration of ${\mathrm{PO}}_{4}^{3-}$, K^+^, Mg^2+^, Na^+^, Cl^−^ was determined by an external laboratory (Demetra S.n.c., Pescia, Italy) on dry stem samples. Particularly, a pool of eight biological replicates was prepared for each treatment and condition. From each pool, three technical sub‐replicates were obtained for MYC and NMYC plants under the SS condition, resulting in a total of six analysed samples. The dry stems were ground into a fine powder to obtain a homogeneous and representative sample for ion analysis.

### 2.7 Statistical Analysis

Statistical analyses were performed using the R software (version 4.1.1). Biometric, gas‐exchange, and biochemical parameters analysed by PCA were subsequently submitted to a 2‐way PERMANOVA analysis using the *adonis2* function from the vegan package in R (Oksanen et al. 2013). For each individual parameter, as well as for ion concentration in the stem, a one‐way ANOVA test was conducted to determine the effect of AMF. ANOVA was performed after verifying the normality and homoscedasticity using the Shapiro–Wilk and Levene's tests, respectively. The standard error of all means was calculated. Mean separation was performed using the Tukey HSD test (*p* value ≤ 0.05). Concerning gene expression, statistical analyses were carried out using the Relative Expression Software Tool REST 2009 v. 2.0.13 (Qiagen; Pfaffl et al. 2002), with a significance threshold of *p* value ≤ 0.05. Bar plots for graphical representation were conducted with GraphPad Prism (version 9). Regarding RNA‐seq analysis, the heatmaps and GO‐enriched plot were elaborated respectively using the *pheatmap* R and *ggplot2/dplyr* packages. The results obtained by RT‐qPCR and RNA‐seq were compared using Pearson's correlation coefficient.

## 3 Results

### 3.1 AMF Colonisation Alters the Whole Plant Physiological Status in Response to Salt Stress

**FIGURE 1:**
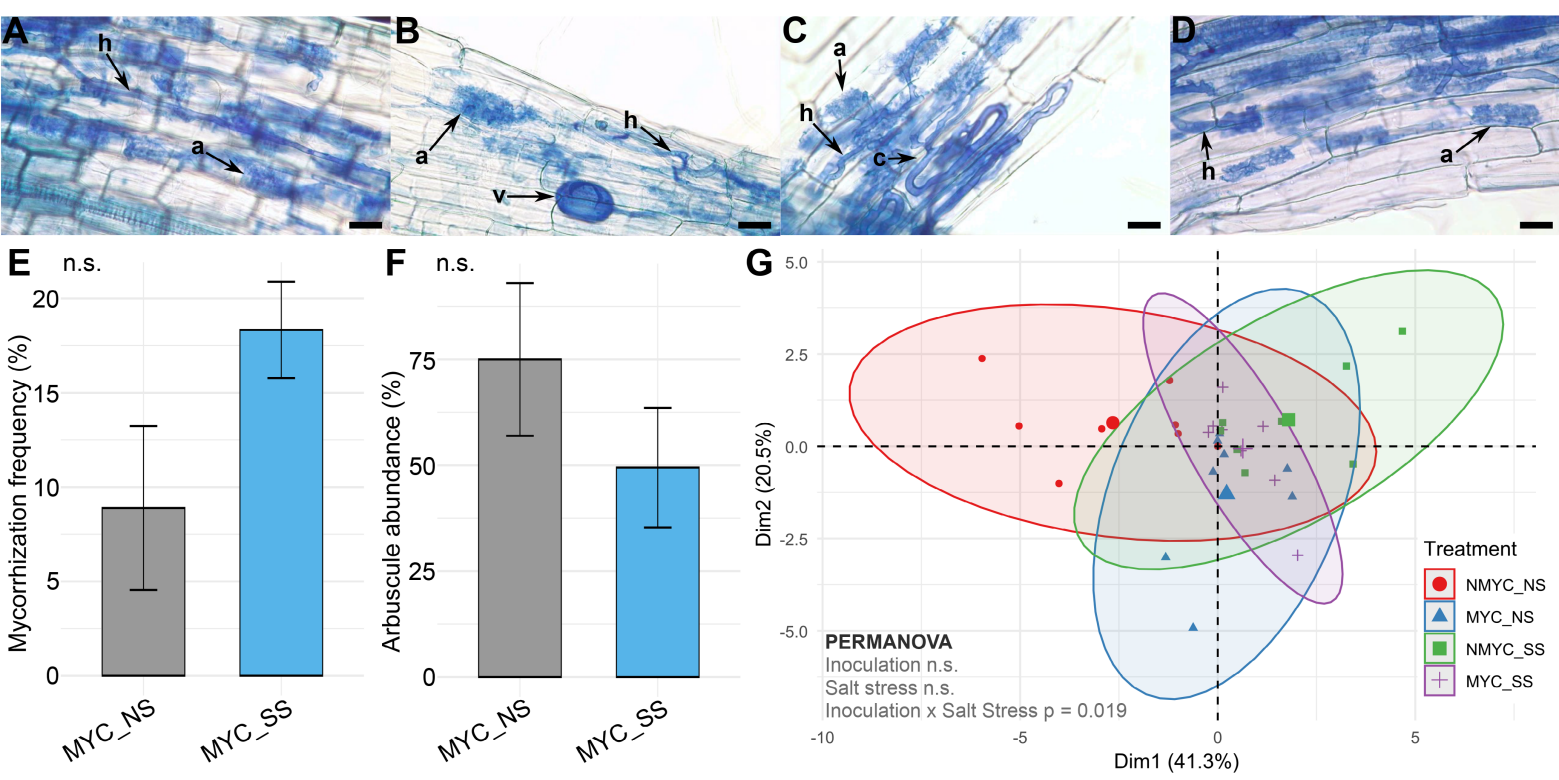
Arbuscular mycorrhizal fungal (AMF) colonization alters overall tomato plant status. (A, B) Representative images of AMF‐inoculated in MYC_NS roots. (C‐D) Representative images of AMF‐inoculated in MYC_SS. MYC plants, both in not‐stressed and salt stress conditions, showed typical AMF structures, including intraradical hyphae (h), hyphal coils (c), arbuscules (a), and vesicles (v). Scale bars = 30 μm. MYC, inoculated; NS, non‐stressed; SS, salt stress. Barplots depict the average of (E) mycorrhization frequency and (F) arbuscule abundance in MYC_NS and MYC_SS roots. Error bars depict standard error (SE). Data was submitted to t‐test statistical analysis. (G) Principal component analysis (PCA) illustrating the overall effect of AM inoculation on plant stress responses (12 biometric, gas‐exchange and metabolic parameters, see M&M). A 2‐way PERMANOVA was performed to find significant effects of inoculation, salt stress or its interaction.

To determine differences in root mycorrhizal colonisation, the mycorrhizal frequency and arbuscule abundance were evaluated in colonised root segments. No colonisation structures were detected in not‐inoculated roots, as previously reported by Giovannini et al. (2024). Both under not‐stressed and salt‐stressed conditions, MYC plants were found colonised and showed typical AM fungal structures such as intraradical hyphae, arbuscules, and vesicles (Figure 1A–D). Although not statistically significant, colonisation frequency under salt stress was almost twice as high (SS ~18% vs. NS ~9%) (Figure 1E), while arbuscule abundance was lower (SS ~50% vs. NS ~75%) (Figure 1F).

To evaluate how AM colonisation influenced the plant's overall response to salt stress, we evaluated plant phenotype under stress, observing yellowish curled leaves in both NMYC_SS and MYC_SS treatments (Figure S1). In addition, certain stress‐related metabolites in leaves were highly accumulated under both salt stress treatments. However, some of them, including pyroglutamic acid, prodelphinidin A1, hydroxycoumarin or fructose, were only found to accumulate in NMYC_SS (Table S3). Finally, we performed a PCA including 12 different biometric, physiological and biochemical parameters in leaves. Neither AM colonisation nor salt stress alone significantly influenced the overall plant physiological status, but their interaction significantly affected it (Figure 1G). By analysing each parameter individually, three different behaviours emerged: (1) basal stem diameter, salicylic acid, abscisic acid and indole acetic acid leaf contents were not significantly affected in any condition (Table 1); (2) CCI and concentrations of proline and jasmonic acid in the leaves were strongly affected by salt stress but unaffected by the colonization status (Table 1); (3) The remaining parameters (i.e., plant height, number of leaves, stomatal conductance, transpiration and net carbon assimilation) followed a similar trend, showing a marked decrease upon salt‐stressed conditions, while this decrease was buffered in AM‐inoculated plants (Table 1).

**TABLE 1:** Biometric, gas‐exchange, and leaf biochemical parameters of AM‐inoculated and not‐inoculated tomato plants in response to salt stress.

	NMYC_NS	NMYC_SS	MYC_NS	MYC_SS
Biometric parameters				
Height (mm)	367.14 ± 19.80a	299.38 ± 17.04ab	262.50 ± 21.70b	258.00 ± 15.66b
N leaves	9.14 ± 0.19a	7.50 ± 0.13b	7.00 ± 0.37b	7.25 ± 0.19b
Stem diameter (mm)	4.68 ± 0.18a	4.13 ± 0.22a	4.26 ± 0.31a	4.10 ± 0.18a
Physiological parameters				
CCI (SPAD)	12.43 ± 0.86a	5.34 ± 0.79b	10.70 ± 0.49a	7.00 ± 0.29b
*g*_s_ (mol H_2_O m^−2^ s^−1^)	0.43 ± 0.03a	0.05 ± 0.02c	0.14 ± 0.02bc	0.21 ± 0.01b
*E* (mmol H_2_O m^−2^ s^−1^)	6.73 ± 0.44a	1.78 ± 0.28c	3.38 ± 0.24b	3.92 ± 0.17b
A (μmol CO₂ m^−2^ s^−1^)	4.83 ± 0.85a	1.42 ± 0.46b	2.28 ± 0.40ab	3.54 ± 0.23ab
Phytohormones and proline content				
SA (ng g^−1^ DW)	2435.47 ± 69.42a	1200.36 ± 591.74a	361.93 ± 287.44a	1459.74 ± 682.18a
JA (ng g^−1^ DW)	18.60 ± 0.25a	7.71 ± 2.46b	17.22 ± 1.29a	2.36 ± 2.74b
ABA (ng g^−1^ DW)	2524.63 ± 986.95a	4722.71 ± 268.44a	2984.01 ± 46.29a	2873.43 ± 845.66a
IAA (ng g^−1^ DW)	353.89 ± 54.17a	407.43 ± 44.80a	258.53 ± 77.23a	406.35 ± 75.71a
Proline (μmol g^−1^ DW)	0.35 ± 0.01b	0.92 ± 0.06a	0.27 ± 0.06b	0.80 ± 0.10a

*Note:* Each value represents the average value of *n* = 8 (biometric parameters), *n* = 4 (gas exchange parameters), and *n* = 3 (phytohormones and proline content) plants per treatment ± standard error (SE). Different letters represent statistical differences across the treatments, according to one‐way ANOVA followed by Tukey's post hoc test. These parameters were obtained in a previous experiment (Giovannini et al. 2024).

Abbreviation: CCI = chlorophyll content index.

### 3.2 Key Salt‐Stress Sensing, Water Transporter, and Antioxidant Enzyme‐Related Genes Exhibit Different Expression Patterns Based on AMF Inoculation

According to the current literature (see materials and methods section), the expression profiles of specific genes from the salt‐stress sensing pathway, together with water transport and the plant antioxidant system, were evaluated. Their transcript abundance was measured in *S. lycopersicum* roots in response to salt stress, with or without AMF‐inoculation.

**FIGURE 2:**
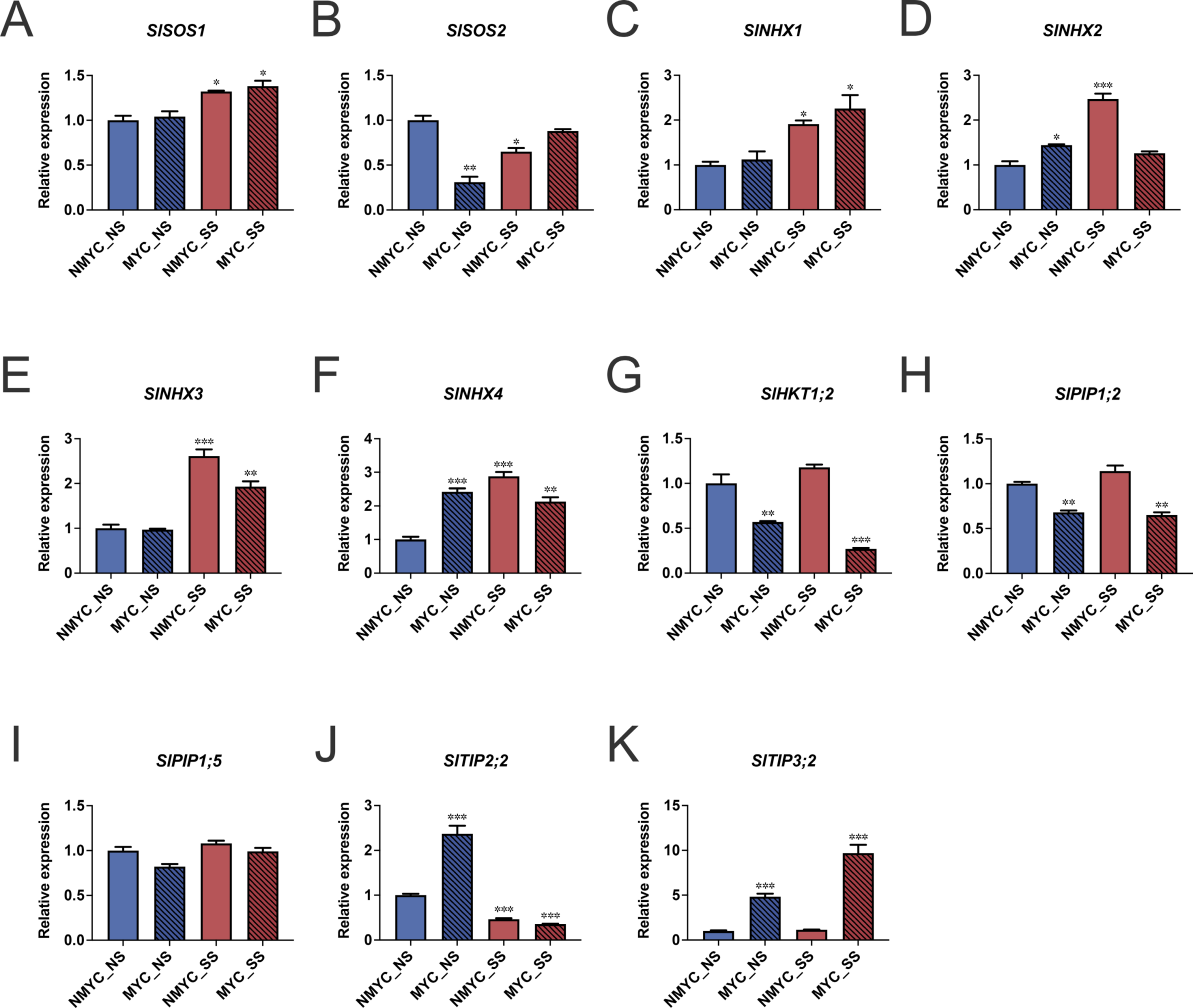
Gene expression profile of key selected Salt Overly Sensitive (SOS) and K^+^, Na^+^, and water transporter genes. For each gene, RT‐qPCR data represent relative expression compared to the NMYC_NS condition, in which the expression was designated to be 1. Values represent the average ± SE (*n* = 3) of each condition. Asterisks represent significant differences (*p < 0.05; **p < 0.01; ***p < 0.001) on each condition compared to NMYC_NS control, according to Relative Expression Software Tool REST 2009 v. 2.0.13. (A) Na^+^/H^+^ antiporter *SlSOS1*; (B) serine/threonine protein kinase *SlSOS2*; vacuolar Na^+^/H^+^ antiporters (C) *SlNHX1*; (D) *SlNHX2*; (E) *SlNHX3*; (F) *SlNHX4*; (G) sodium transporter *SlHKT1*; aquaporins (H) *SlPIP1;2*; (I) *SlPIP1;5*; (J) *SlTIP2;2*; (K) *SlTIP3;2*.

**FIGURE 3:**
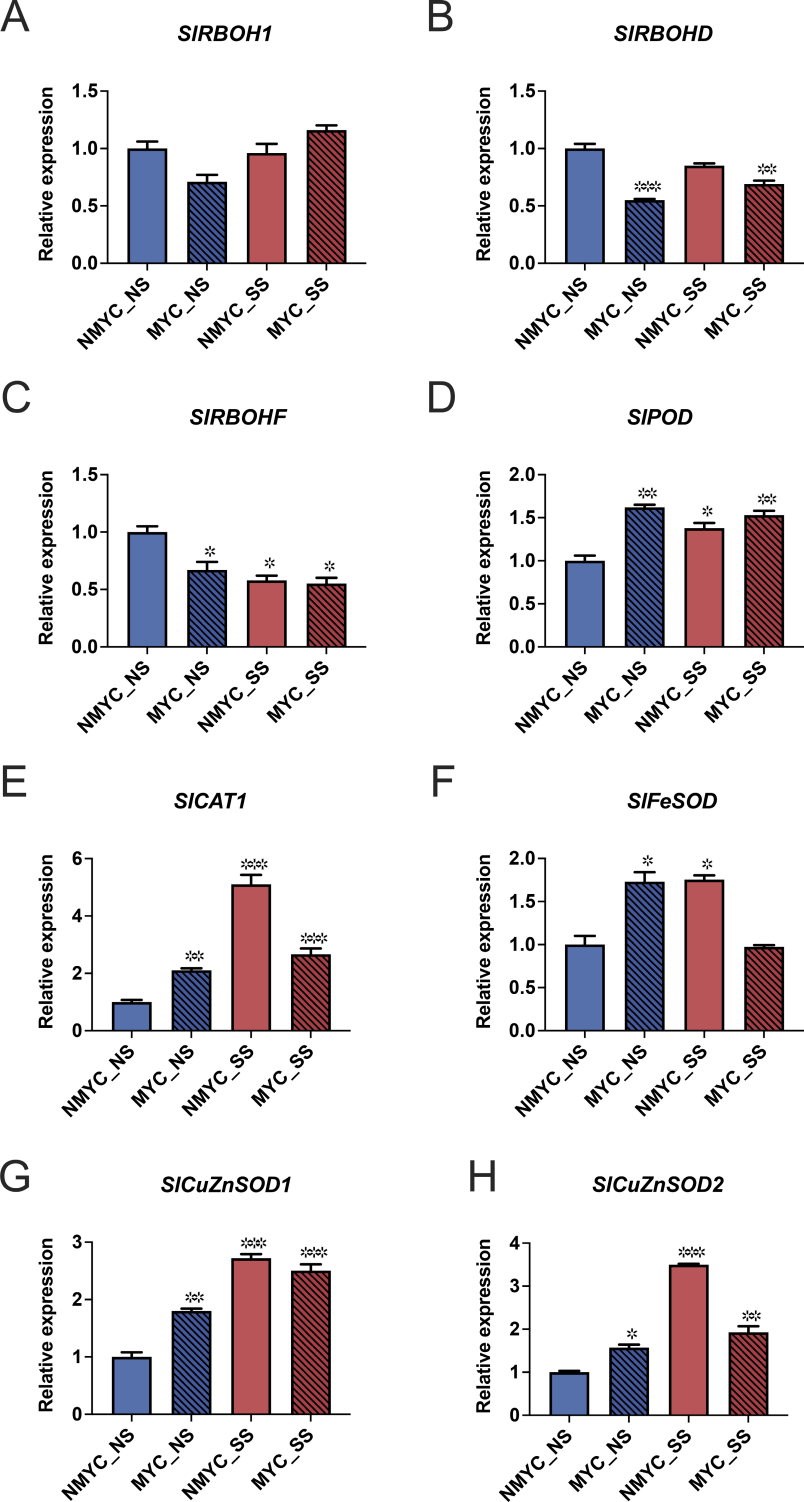
Gene expression profile of key antioxidant genes. For each gene, RT‐qPCR data represent relative expression compared to the NMYC_NS condition, in which the expression was designated to be 1. Values represent the average ± SE (*n* = 3) of each condition. Asterisks represent significant differences (*p < 0.05; **p < 0.01; ***p < 0.001) on each condition compared to NMYC_NS control, according to Relative Expression Software Tool REST 2009 v. 2.0.13. Respiratory Burst Oxidase Homologs (A) *SlRBOH1*; (B) *SlRBOHD*; (C) *SlRBOHF*; Peroxidase *SlPOD* (D); Catalase *SlCAT1* (E); Superoxide dismutases (F) *SlFeSOD*; (G) *SlCuZnSOD1*; (H) *SlCuZnSOD2*.

Regarding the salt stress sensing pathway, *SlSOS1* was upregulated under SS conditions, regardless of the mycorrhization status (Figure 2A and Table S4), whereas *SlSOS2* was downregulated in MYC_NS and NMYC_SS, but not in MYC_SS (Figure 2B and Table S3). *SlNHX* vacuolar Na^+^/H^+^ exchangers showed an overall upregulation under salt stress (except for *SlNHX2*), both in MYC and NMYC conditions. In addition, *SlNHX2* and *SlNHX4* were also upregulated in MYC_NS conditions (Figure 2C–F and Table S4). Finally, the *SlHKT1;2* high‐affinity K^+^ channel was strongly downregulated under AM colonisation, with this effect more significant in SS than in NS conditions (Figure 2G and Table S4). When focusing on water and solute transporters, that is, genes coding for several *AQPs*, different patterns of expression were found. AM inoculation downregulated the expression of *SlPIP1;2* and greatly upregulated *SlTIP3;2* expression in roots, both in NS and SS conditions (Figure 2H,K and Table S4). In the case of *SlTIP3;2*, this gene was up to two times more expressed in MYC_SS (10‐fold increase) than in MYC_NS (5‐fold increase) conditions. *SlTIP2;2* was upregulated in MYC_NS conditions, but downregulated under SS, regardless of the mycorrhization status (Figure 2J and Table S4). Not significant regulation of *SlPIP1;5* was found (Figure 2I and Table S4). Finally, we checked the transcriptional status of the antioxidant system in tomato roots. *SlRBOHs* were either unaffected (*SlRBOH1*, Figure 3A and Table S4) or significantly downregulated in response to AM inoculation, both under NS and SS conditions (*SlRBOHD* and *SlRBOHF*, Figure 3B,C, respectively and Table S4). The expression of most of the evaluated antioxidant enzymes‐encoding genes was significantly higher under all conditions with respect to the NMYC_NS controls, including *SlPOD* (Figure 3D), *SlCAT1* (Figure 3E), *SlCuZnSOD1* (Figure 3G and Table S4) and *SlCuZnSOD2* (Figure 3H and Table S5). Specifically, for *SlCAT1*, *SlFeSOD* (Figure 3F and Table S5) and *SlCuZnSOD2*, the observed upregulation was significantly higher in NMYC_SS than in MYC_SS.

### 3.3 AMF Inoculation Perturbs the Whole Root Transcriptome in Response to Salt Stress

**FIGURE 4:**
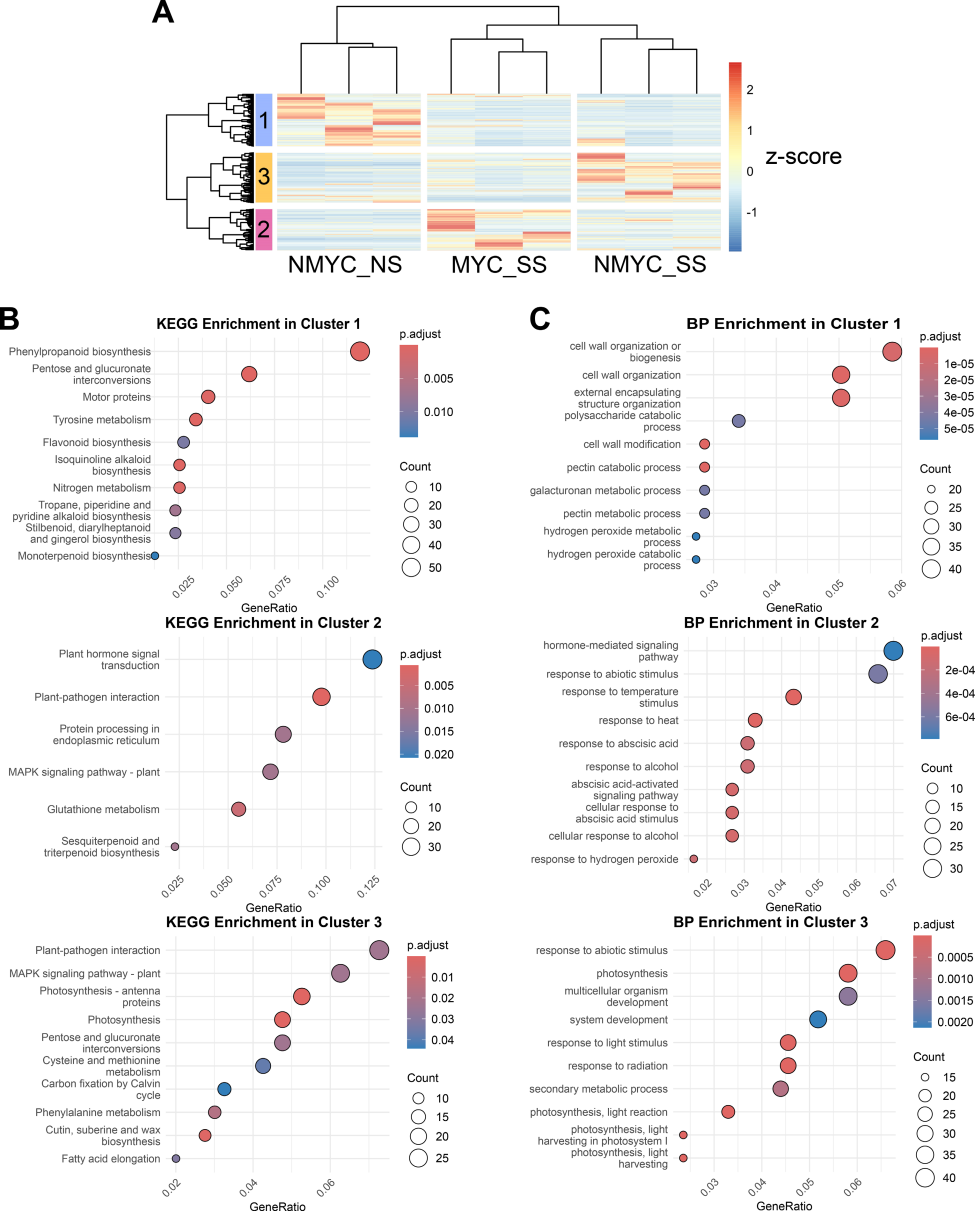
Transcriptional landscape of arbuscular mycorrhizal inoculated (MYC) or non‐inoculated (NMYC) *Solanum lycopersicum* roots under salt‐stress (SS). (A) Heatmap representation of the Z‐score DESEQ2 normalized reads of the 14,076 most variable genes in the tomato root RNA‐seq dataset. Hierarchical clustering was performed using Euclidean distance as the clustering metric and the Ward.D2 method for linkage. Rows and columns were grouped into three clusters each. The row annotations indicate gene clusters, with colours and numbers corresponding to cluster groups. (B) Kyoto Encyclopedia of Genes and Genomes (KEGG) enrichment analysis of gene clusters. Each graph shows the top 10 highest significantly enriched (Padj < 0.05) pathways for each cluster. Dot size represents the count of genes presenting each KEGG annotation and colour represents significance level. (C) Gene Ontology (GO) enrichment analysis of gene clusters. Only Biological Processes (BP) terms were analyzed. Each graph shows the top 10 highest significantly enriched (Padj < 0.05) terms for each cluster. Dot size represents the count of genes presenting each term annotation and colour represents significance level.

The observed alterations in key genes of salt stress sensing and response prompted us to inspect the whole transcriptional landscape of tomato roots under salt stress. First, we checked the expression of the previously studied genes by RT‐qPCR, finding a statistically significant positive correlation between these data and RNA‐seq data (*r* = 0.76, *p* < 0.0001, Figure S2). However, minor discrepancies (i.e., *SlSOS2* or *SlNHX2*) were also found. We generated 13.28 million paired reads *per* sample on average, with mapping scores around 90% to the *Solanum lycopersicum* SL3.1 draft genome (Table S5). In total, we found 29,996 expressed tomato genes in our dataset, which exhibited differences between how mycorrhizal roots respond to salt stress compared to non‐mycorrhizal roots (Figure S4). After filtering out low variable genes (Coefficient of variation < 0.5), 14,076 genes were retained, which were grouped in three main clusters according to hierarchical clustering based on their normalised gene expression profile (Figure 4A and Table S6). Those genes with a trend towards downregulation under salt stress, regardless of the mycorrhizal status, were grouped in cluster 1 and showed enrichment in KEGG terms related to phenylpropanoid or flavonoid biosynthesis and GO biological processes (BP) related to cell wall modification. Cluster 2 comprised genes generally upregulated in MYC_SS but not in NMYC_SS and exhibited enrichment of GO terms related to plant hormone regulation (such as abscisic acid) or MAPK signalling, as well as response to plant pathogens and abiotic stimulus or response to hydrogen peroxide. Cluster 3 showed the opposite trend: upregulation in NMYC_SS but not in MYC_SS. This latter cluster shared certain enriched terms with cluster 2, including plant‐pathogen interaction, response to abiotic stimulus or MAPK signalling (Figure 4B).

**FIGURE 5:**
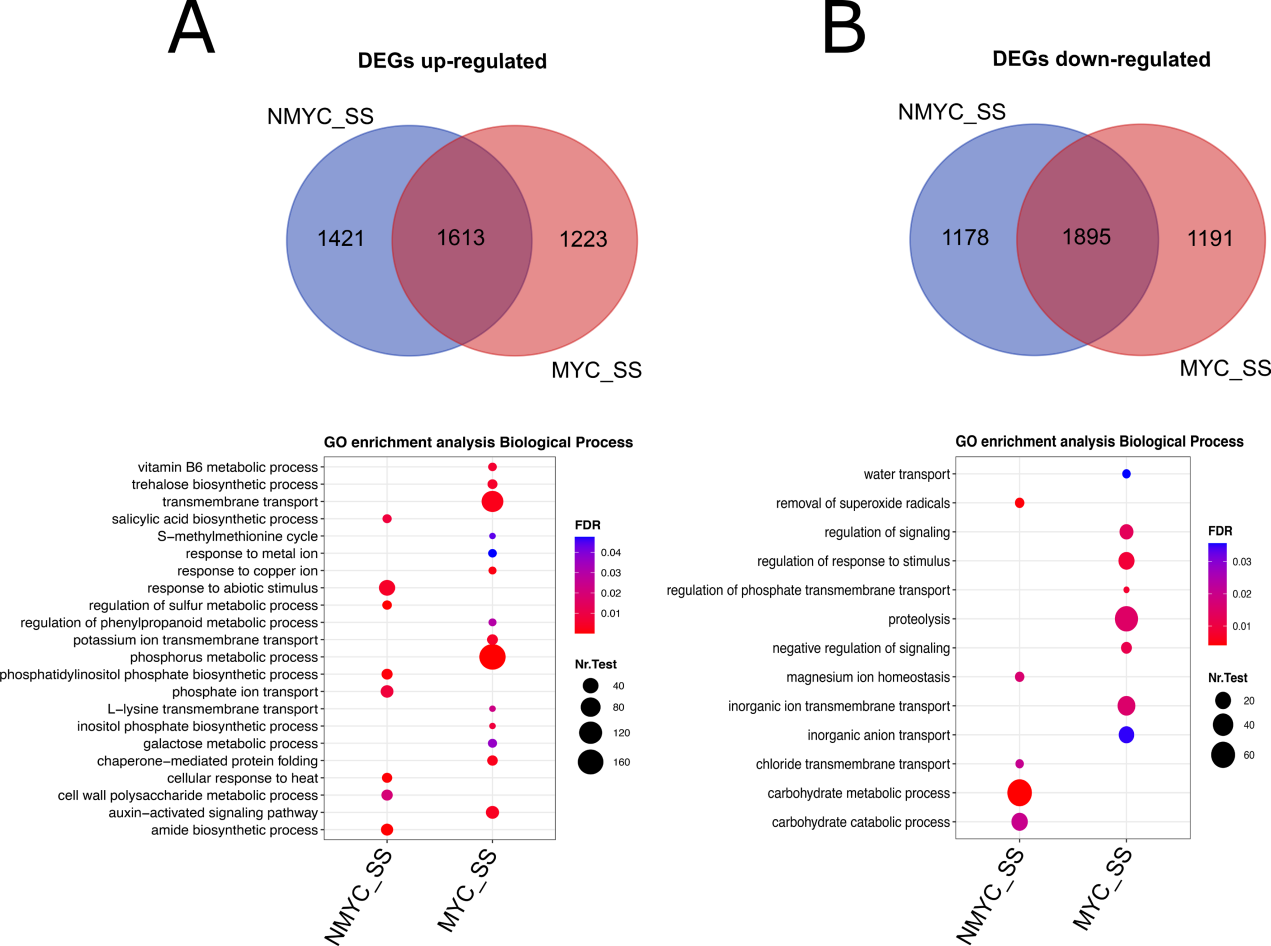
Unique and overlapping genes and biological processes differentially regulated by non‐inoculated and AMF‐inoculated roots under salt stress. At the top part of the image, Venn's diagram showing unique and common up (A) and down (B) regulated genes in MYC_SS versus NMYC_NS and NMYC_SS versus NMYC_NS conditions. At the bottom, gene ontology (GO) enrichment analysis of unique up (A) and down (B) regulated genes. Only Biological Processes (BP) terms were analysed. Dot size represents the count of genes presenting each GO term annotation and colour represents significance level.

Differential expression analysis (Figure S5) of NMYC_SS and MYC_SS compared to NMYC_NS allowed identifying a total of 8384 unique DEGs (Tables S7–S9). NMYC_SS roots showed 3033 upregulated and 3072 downregulated genes, respectively, when compared to NMYC_NS (FDR < 0.05; Table S8). On the other side, roots of MYC_SS plants had 2835 upregulated and 3085 downregulated genes, respectively, when compared to NMYC_NS (FDR < 0.05; Table S9). To evaluate the differences in salt‐stress sensing and response between NMYC_SS and MYC_SS roots, we overlapped DEGs in these two conditions (Figure 5). We found 1613 commonly upregulated genes, while 1421 were exclusively upregulated in NMYC_SS and 1223 in MYC_SS (Figure 5A, Tables S10 and S11). On the other hand, we found 1895 commonly downregulated genes, while 1178 were only downregulated in NMYC_SS and 1191 in MYC_SS (Figure 5B, Tables S12 and S13). GO‐enrichment of the exclusively up‐ and down‐regulated genes showed different enriched terms related to BPs in the two groups. A total of 14 GO terms related to BPs were found in MYC_SS, including phosphorus metabolic process or transmembrane transport. Eight BPs were exclusive of NMYC_SS roots, highlighting response to abiotic stimulus (Figure 5A). On the other side, 8 BPs were enriched in downregulated genes in MYC_SS roots, including several terms related to ion and water transport, signalling and proteolysis. Finally, 5 BPs were significantly enriched among the genes down‐regulated in NMYC_SS, most of which are related to carbohydrate‐metabolic processes (Figure 5B).

Among the top exclusively regulated genes in MYC_SS (Table S11), it is worth noting that we found key genes belonging to the Common Symbiosis Pathway and related to mycorrhizal functioning, such as phosphate transporter *SlPT4*, ammonium transporter *SlAMT2*, and to the lipid biosynthesis and transport, including *SlRAM2*, *SlSTR2* and *SlFatM*. The expression of these genes remained unchanged in NMYC_SS plants (Figure S6). Other transcripts mainly involved in signal transduction, the SOS pathway, ion homeostasis and antioxidant responses were also differentially regulated between NMYC_SS and MYC_SS roots.

#### 3.3.1 Signal Transduction

**FIGURE 6:**
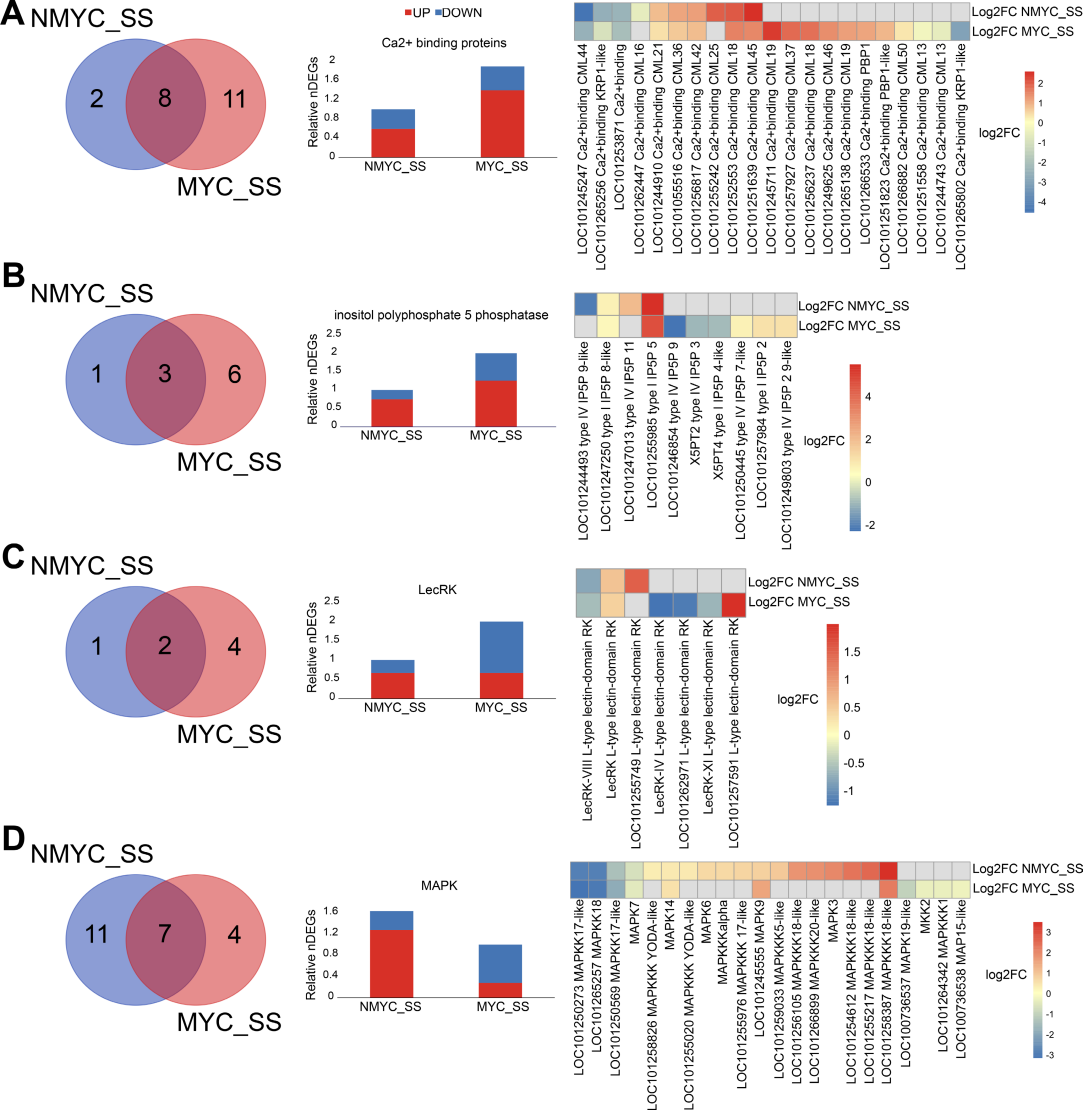
Non‐inoculated and AMF‐inoculated tomato roots differentially regulate signal transduction‐related genes. From left to right, Venn's diagram, barplot, and heatmap depicting the number and level of expression of (A) Ca^2+^ binding proteins, (B) inositol polyphosphate 5′ phosphatases, (C) lectin receptor kinases, and (D) mitogen activated protein kinases.

Several terms related to signal transduction were enriched in salt‐stress conditions (Figures 4 and 5), and, therefore, we decided to have a deeper look into this function. We found a total of 645 DEGs, including putative genes related to protein phosphatases and phospholipases, kinases or calcium‐signalling related genes (Table S14). Among them, 273 were commonly regulated in NMYC_SS and MYC_SS roots. However, MYC_SS plants showed a slightly larger set of uniquely regulated genes than NMYC_SS plants (208 vs. 164 DEGs), resulting in 1.1‐fold higher DEGs of signal transduction functions in MYC_SS than in NMYC_SS (Figure S7). Although the overall increase was moderate, certain putative families showed much marked differences than others. Ca^2+^ binding proteins (mainly calmodulin like), inositol phosphatases and L‐type lectin receptor kinase DEG counts were between 1.9 and 2.33‐fold higher in MYC_SS than in NMYC_NS conditions (Figure 6A–C). On the other hand, mitogen‐activated protein kinase (MAPK) DEG count showed an inverse trend, with a 1.63‐fold increase of DEGs in NMYC_SS than in MYC_SS (Figure 6D).

#### 3.3.2 Salt Overly Sensitive Pathway, K^+^ Transport and Ion Homeostasis

**FIGURE 7:**
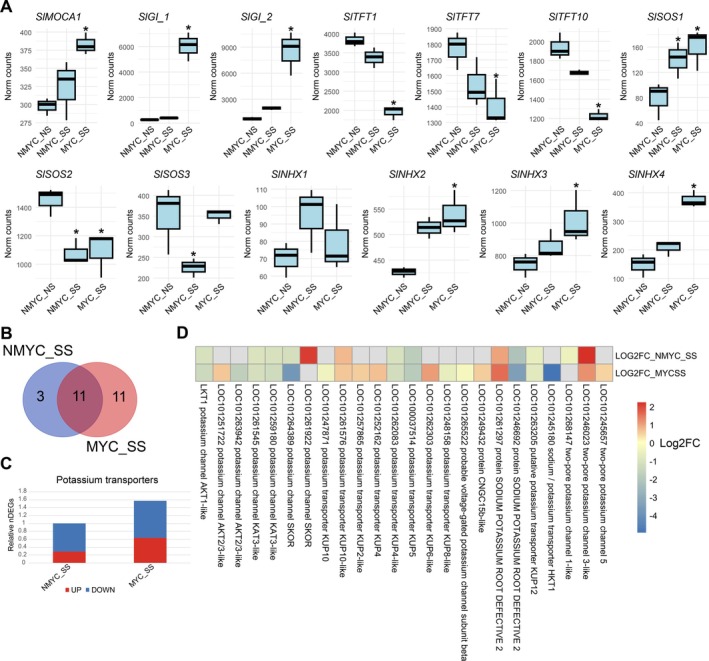
Non‐inoculated and AMF‐inoculated tomato roots differentially regulate salt overly sensitive and potassium transport genes. (A) Boxplot represent the DESeq2 normalized counts from salt‐stress sensing and salt overly sensitive (SOS) pathway. Whiskers represent the limits of the 1.5 interquartile range. Statistical differences are represented with an asterisk, based on DEseq2 analysis (FDR < 0.05). (B) Venn's diagram depicting the number of unique and overlapping differentially expressed genes (DEGs) encoding for potassium transporters between NMYC_SS and MYC_SS. (C) Barplot showing the relative number of potassium transporters DEGs in NMYC_SS and MYC_SS conditions. (D) Heatmap representing Log2FC of potassium transporters.

**FIGURE 8:**
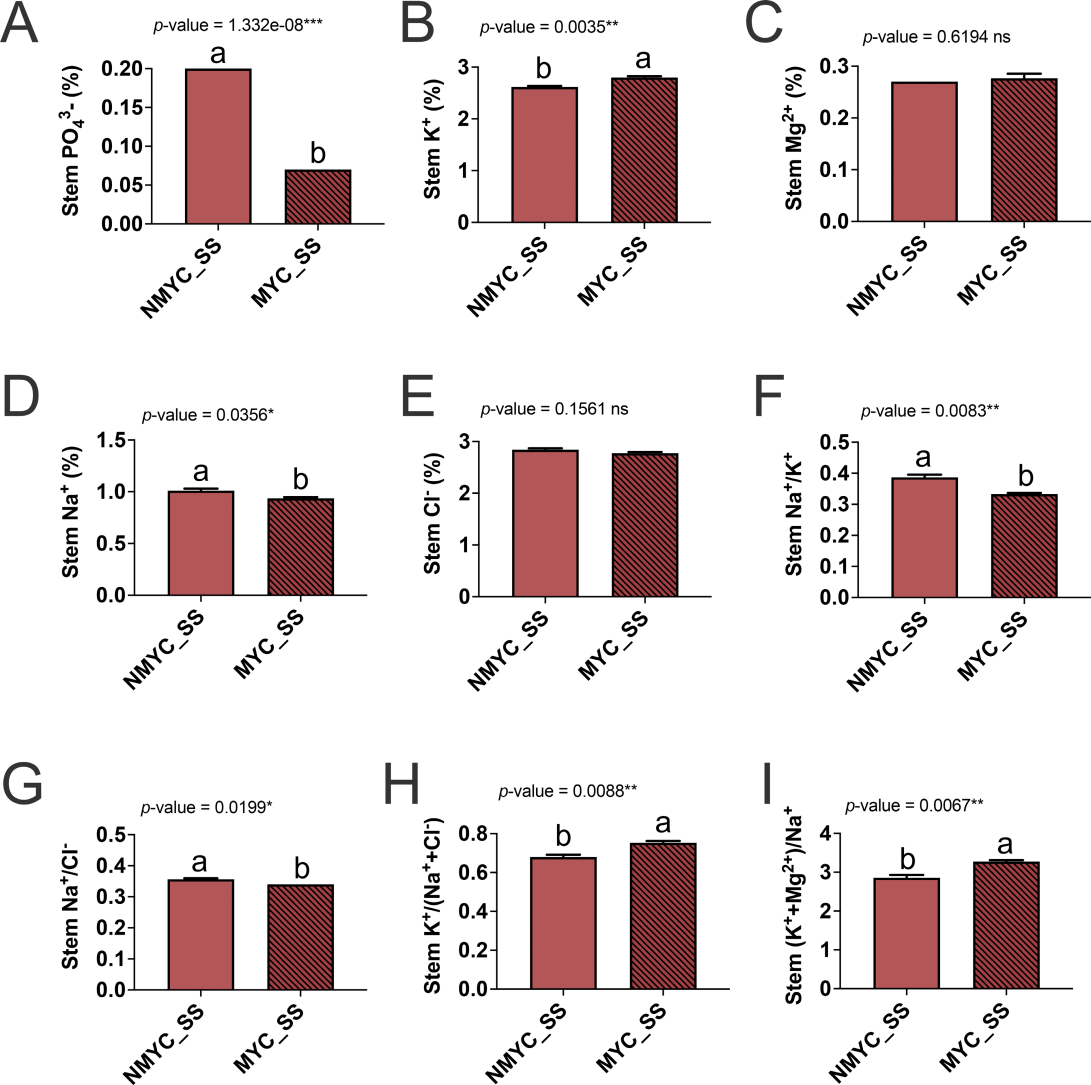
AMF‐inoculation alters ion concentrations in tomato stems under salt stress. Barplots representing the relative concentrations (%) of different ions and their ratios in tomato stems under salt stress. Different letters indicate significant differences (*p* < 0.05) between NMYC_SS and MYC_SS treatments.

Due to its crucial role in salt‐stress sensing and response, we had a deeper look into the SOS pathway regulation of NMYC and MYC roots. *SlTFTs* and *SlGIs* encode negative regulators of the SOS pathways and were differently regulated in MYC_SS and NMYC_SS roots. *SlMOCA1*, an inositol phosphorylceramide glucuronosyltransferase involved in salt‐stress sensing, was slightly upregulated in MYC_SS, while it was unaffected in NMYC_SS roots (Figure 7A). While three different *SlTFT* genes (*TFT1, TFT7* and *TFT10*) were uniquely downregulated in MYC_SS, two different *SlGI* genes (LOC101258346 and LOC101255788) were upregulated in MYC_SS roots (Figure 7A). *SlSOS1* was found to be significantly upregulated in both cases, while *SlSOS2*, on the other hand, was downregulated (Figure 7A). *SlSOS3* was found to be downregulated solely in NMYC_SS roots, while its expression was maintained in MYC_SS roots (Figure 7A). Finally, *SlNHX2‐4* showed a similar trend. They were significantly upregulated in MYC_SS but not in NMYC_SS, although a trend towards upregulation was also found. *SlNHX1* was much less abundant than the others, and no significant differences were observed (Figure 7A). Overall, we noticed an increased number of DEGs (1.56‐fold) related to potassium and sodium transport in MYC_SS compared to NMYC_SS roots (Figure 7B,C). While different potassium transporters, such as *AKT1* genes, were equally regulated in both conditions, others were differentially expressed between MYC_SS and NMYC_SS conditions (Figure 7D). Two different *SKOR* genes were differentially regulated, since LOC101264389 was more strongly downregulated in AMF‐inoculated roots than in non‐inoculated roots, while another gene (LOC101261922) was upregulated in non‐inoculated roots, but not significantly regulated in AMF‐inoculated roots (Figure 7D). Two *AKT2/3* genes were exclusively regulated in AMF‐inoculated roots in response to salt stress, although one was slightly upregulated (LOC101251722) and the other downregulated (LOC101263942) (Figure 7D). Potassium uptake proteins from the KUP family were also differentially regulated, with *KUP2/4/6* upregulated and *KUP8/10* downregulated, in both cases exclusively in AMF‐inoculated roots (Figure 7D). Finally, the High‐Affinity K^+^ Transporter *SlHKT1* was highly downregulated exclusively in MYC_SS roots under salt stress (Figure 7D). Considering these results, we decided to test if the observed transcriptional trends resulted in the alterations of *in planta* physiological levels of different ions at the stem level, including ${\mathrm{PO}}_{4}^{3-}$, Na^+^, K^+^, Mg^2+^ and Cl^−^ (Figure 8 and Table S15). Stem tissue was chosen to assess ion content to better integrate ion uptake and accumulation, as well as to evaluate ion transport. We found a significant 2.86‐ and 1.08‐fold decrease of ${\mathrm{PO}}_{4}^{3-}$ and Na^+^ contents, respectively, in MYC_SS compared to NMYC_SS plants. On the other hand, we found a significant 1.07‐fold increase in K^+^. Mg^2+^ and Cl^−^ levels remained constant. Not only the ion content, but also the ratio between different ions was affected by AM inoculation. While Na^+^/K^+^ and Na^+^/Cl^−^ ratios decreased, K^+^/(Na^+^ + Cl^−^) and (K^+^ + Mg^2+^)/Na^+^ increased (Figure 8 and Table S15).

#### 3.3.3 Response to Reactive Oxygen Species

**FIGURE 9:**
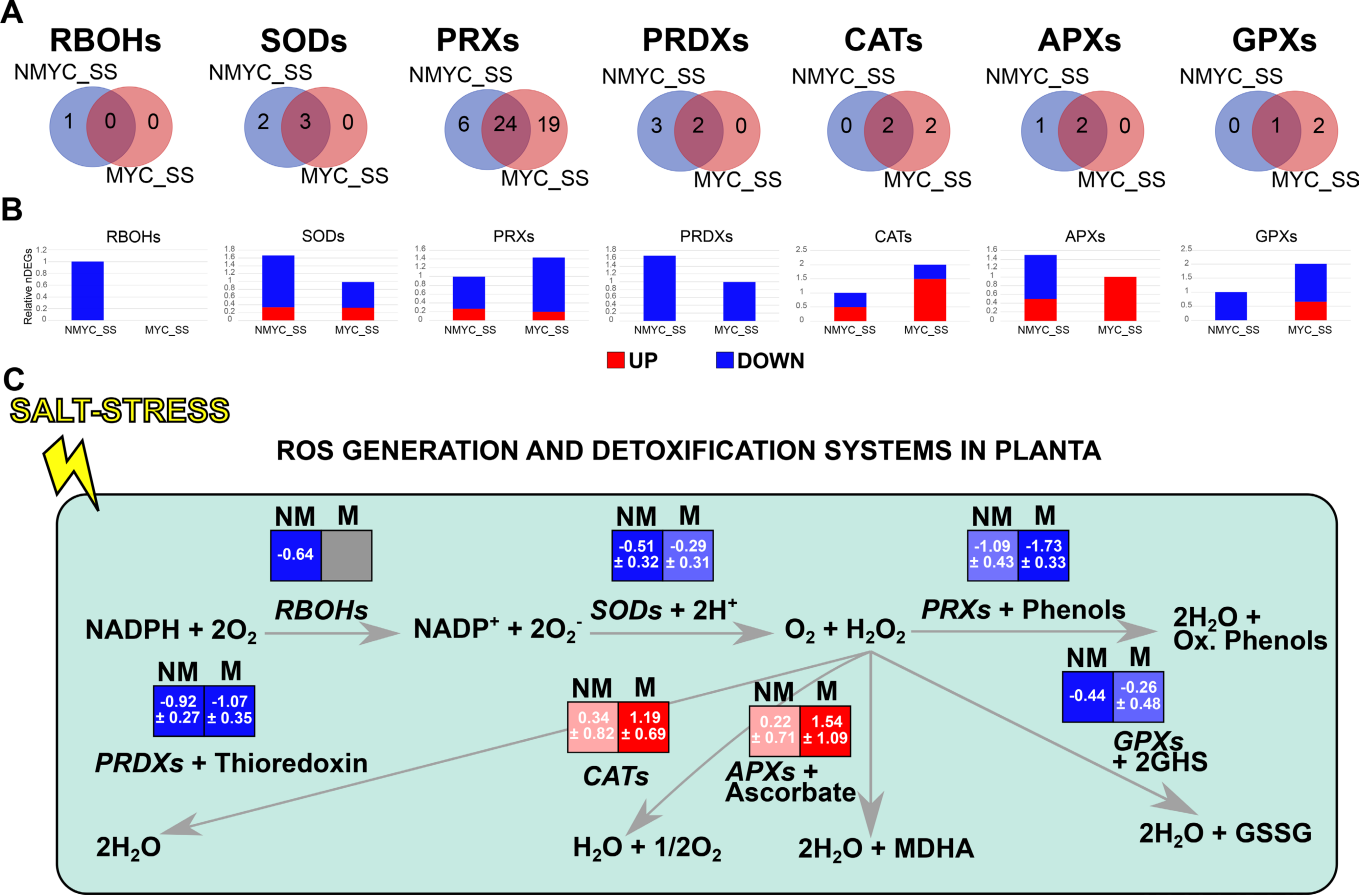
Non‐inoculated and AMF‐inoculated tomato roots differentially regulate antioxidant genes. (A) Venn's diagrams of unique and overlapping differentially expressed genes (DEGs) encoding for respiratory burst oxidase homologs (RBOHs), superoxide dismutases (SODs), peroxidases (PRXs), peroxiredoxins (PRDXs), catalases (CATs), ascorbate peroxidases (APXs) and glutathione peroxidases (GPXs) between NMYC_SS and MYC_SS roots. (B) Barplot showing the relative number of these same gene families in NMYC_SS and MYC_SS conditions. (C) Schematic representation of the reactive oxygen species (ROS) generation and detoxification in planta. The mean Log2FC value ± standard error for all the DEGs in each family is represented in the coloured squares. Blue indicates downregulation and red upregulation. GSSG = glutathione disulfide; M = mycorrhizal; MDHA = Monodehydroascorbate; NM = non‐mycorrhizal; Ox. = oxidized.

Several terms related to hydrogen peroxide metabolism and superoxide radicals were found in the performed enrichment analyses (Figures 4 and 5). Therefore, we investigated this important function of salt‐stress response in our RNA‐seq dataset. Regulation of several genes encoding key antioxidant enzymes was found (Table S15). NMYC_SS roots regulated a higher number of respiratory burst oxidase homologs (RBOHs), superoxide dismutases (SODs), peroxiredoxins (PRDXs) and ascorbate oxidases, while MYC_SS roots regulated a higher number of peroxidases (PRXs), catalases (CATs) and glutathione peroxidases (GPXs) (Figure 9A,B). In any case, most of these regulated genes were downregulated, highlighting PRXs with a 1.45‐fold higher number of DEGs in MYC_SS than in NMYC_SS. Despite this, a peroxidase family 44 (LOC101247989) was found to be among the top 50 most upregulated genes in MYC_SS conditions (Log2FC = 6.46, FDR = 0.0006066) and was not regulated in NMYC_SS roots. CATs and APXs were the only antioxidant‐encoding genes generally upregulated under salt stress. In both these cases, the average Log2FC of these DEGs was higher in MYC_SS than in NMYC_SS roots (Figure 9C). Genes annotated with other functions potentially involved in redox homeostasis that were significantly enriched in our RNA‐seq data, such as flavonoid metabolism, were mainly downregulated during salt‐stress response, while glutathione metabolism or heat stress response factors were mainly upregulated. However, no striking differences between NMYC_SS and MYC_SS were found (Table S16).

## 4 Discussion

Arbuscular mycorrhizal (AM) symbiosis‐induced benefits against the detrimental effects of salt stress are well‐documented in plants, and they range from improved ionic balance, antioxidant production or photosynthesis and water‐use efficiency, among others (Evelin et al. 2019). We have recently demonstrated that *F. mosseae* inoculation, alone or coupled with seed priming, modifies leaf plant responses both to water‐ and salt‐stress in tomato by buffering stomatal conductance decreases and leading to the accumulation of specific primary and secondary metabolites (Giovannini et al. 2024). In the present work, we found that the main physiological parameters negatively affected by salt stress were buffered by AM inoculation, such as gas‐exchange rates. These results reinforce the current knowledge of AM inoculation as an effective tool to partially counter the negative effects of salt stress in agriculture (Phour and Sindhu 2024). Linked to these benefits, we have studied the differential transcriptional responses of AM‐inoculated and not‐inoculated tomato plants under salt stress at the root level. It is noteworthy that, despite the overall good correlation between the RT‐qPCR and transcriptomic data, some discrepancies were found. These minor discrepancies have been previously reported (Everaert et al. 2017) and are concentrated mainly in low differentially expressed genes (Log2FC < 2). Our findings show that AMF inoculation improves ion homeostasis by increasing K^+^ and decreasing Na^+^ ion contents in the stem, in line with previous reports in tomato (Kong et al. 2020) and other species (Giri et al. 2007; Evelin et al. 2012; Porcel et al. 2016; Chen et al. 2017; Wang et al. 2021). This AMF‐mediated salt stress tolerance is transcriptionally linked with the regulation of Ca^2+^ signalling, the SOS pathway, K^+^ transporters and antioxidant enzymes in the roots.

Plants respond to abiotic and biotic stresses through a wide array of physiological, molecular and cellular adjustments. Ca^2+^ ion is well known for its role as a secondary messenger and mediator of plant signalling in many processes (Thor 2019). Ca^2+^‐dependent signalling plays a significant role in plant salt‐stress responses (Manishankar et al. 2018; Bachani et al. 2022). More specifically, calmodulins decode calcium signatures to modulate downstream responses (Chin and Means 2000), while inositol phosphatases are involved in Ca^2+^ mobilisation and regulation (Berridge and Irvine 1989). Particularly, these last have already been associated with the plant response to salinity (Nelson et al. 1998; Jia et al. 2019; Feng et al. 2025). AMF‐inoculation increased the number of calmodulins and inositol phosphatases regulated in response to salt stress, which may contribute to a fine‐tuned, faster and stronger response. It is worth noting that Ca^2+^ signalling also plays a crucial role in the molecular mechanism of symbiosis and is a core part of the ancient signalling pathway known as the Common Symbiotic Pathway (CSP) (Oldroyd 2013). Therefore, the increased number of regulated calmodulins and inositol phosphatases could be attributed to the fact that AMF‐inoculated plants are dealing at the same time with salt stress and AM formation. However, AMF are well‐known to prime plants for better stress tolerance (Lenoir et al. 2016). Thus, our data suggest that the inositol phosphatase‐calmodulin axis may act as a molecular bridge between AM formation and salt‐stress response and could be involved in the crosstalk between symbiosis and salt stress signalling pathways. Further studies are needed to decipher whether a synergy really exists between AMF inoculation and salt stress and to further investigate the role of these genes in the mycorrhizal priming against abiotic stresses.

Under exposure to high [NaCl], plant cells increase Ca^2+^ cytoplasmic concentrations, which is decoded by the SOS pathway, resulting in the exclusion of toxic Na^+^ out of the cytoplasm, thus improving ion homeostasis and salt tolerance (Yang and Guo 2018a, 2018b). MOCA1, a glucuronosyltransferase for glycosyl inositol phosphorylceramide (GIPC) sphingolipids in the plasma membrane of *Arabidopsis thaliana*, is required for salt‐induced depolarisation of the cell‐surface potential, Ca^2+^ spikes and waves, Na^+^/H^+^ antiporter activation, and regulation of growth under salt stress (Jiang et al. 2019). We found that AMF‐inoculated tomato roots increased the regulation of *SlMOCA1* (LOC101249825), the homolog of the *Arabidopsis* gene encoding MOCA1, thus contributing to an improved salt‐sensing mechanism. Not only this, but also different parts of the canonical SOS pathway were affected by AMF inoculation. The impact of AMF inoculation of tomato roots on the expression of SOS pathway genes has been previously reported (Liu et al. 2023). In our experimental setup, all *SlTFT*s were slightly downregulated in non‐inoculated roots but much more strongly downregulated in AMF‐inoculated roots. This can be interpreted as an AMF‐induced improvement of salt stress signalling, as a greater downregulation of TFT genes, which have been recently characterised in tomato and are negative regulators of SOS2 (Jia et al. 2022), may help to maintain the SOS pathway active during salt stress. However, *SlGI* genes, which are also negative regulators of SOS2 (Park et al. 2013), showed the opposite trend, being strongly upregulated in AMF‐inoculated roots. Despite further investigation being required to clarify this finding, a possible explanation could be the presence of a negative feedback to avoid excessive activation of the SOS pathway, as it occurs in other signalling pathways (Jindal et al. 2022). As stated above, the final output of the SOS pathways consists of the activation of transporters, excluding Na^+^ from the cytoplasm. SOS1, a membrane Na^+^/H^+^ antiporter (Shi et al. 2000) and the family of NHX vacuolar Na^+^/H^+^ antiporters (Yokoi et al. 2002) are in charge of this task. Overall, our data support the idea that AMF‐inoculated roots exclude Na^+^ out of the cytoplasm with more efficiency, by upregulating membrane (to a lesser extent) and vacuolar (to a higher extent) Na^+^/H^+^ antiporters, thus contributing to an enhanced salt stress tolerance. In the particular case of the NHX family, although all members were shown to play an important role in salt stress tolerance in diverse species (Yarra 2019), our data suggest that the function of *SlNHX1* seems to be the least relevant, as it shows low levels of expression and is not regulated in any condition. Nonetheless, further experimental studies considering different layers of regulation should be set up to understand possible redundancies and/or specificities within this gene family.

K^+^ retention in the cytosol is an important mechanism mediating salt tolerance, acting directly in ion homeostasis by the improvement of Na^+^/K^+^ ratios but also as a signal molecule (Wu et al. 2018). Although a general regulation of genes related to K^+^ transport was found for both AMF‐inoculated and non‐inoculated roots, the number of regulated transporters was more than 50% higher for AM roots. Both the shaker‐like K^+^ outward rectifying channel (SKOR) and the bidirectional K^+^ weakly inward‐rectifying channels (AKT2/3) are involved in root‐to‐shoot K^+^ translocation (Gaymard et al. 1998; Marten et al. 1999). In our experimental setup, the genes coding for these channels were either upregulated (such as the *SKOR* gene LOC101261922) or not regulated in non‐inoculated roots under salt stress, in agreement with previous reports (Maathuis 2006). Our data suggest that AMF inoculation alters this trend, since these same genes change from up or no regulation in non‐inoculated conditions, to slight or severe downregulation in AMF‐inoculated roots (for instance, the *SKOR* gene LOC101264389). Previous reports showed a decline in the root‐to‐leaf gradient of Na^+^/K^+^ ratio in maize associated with AMF inoculation (Wang et al. 2021), which is in accordance with the expression profile here reported. On the other hand, the high‐affinity K^+^ transporter *SlHKT1*, despite its name, plays a significant role in mediating tolerance to salt stress by unloading Na^+^ from xylem vessels to parenchyma cells (Sunarpi et al. 2005). HKT1 is considered a key determinant of plant salinity tolerance (Hauser and Horie 2010). In fact, *HKT1* mutations suppress the salt‐hypersensitive phenotypes in both sos2 and sos3 mutants, suggesting that HKT1 coordinates with the SOS pathway to modulate Na^+^/K^+^ homeostasis in plant cells (Rus et al. 2004). The downregulation of *SlHKT1* observed in AMF‐inoculated roots could be a response to the lower Na^+^ concentration observed in these plants. Overall, our results suggest that AMF influence the regulation of K^+^ and Na^+^ transporters in the roots. All the observed differences are related to the improvement of K^+^ retention and the exclusion of Na^+^, which concurs with the improved Na^+^/K^+^ ratios observed in our experiment. However, the exact role of each of these genes cannot be determined merely by transcriptomics and further studies are required to explore their localisation, other mechanisms of regulation, such as posttranslational modifications, and specific roles in AMF‐mediated salt tolerance.

Although the antioxidant response was similar under salt stress, we observed transcriptional alterations in AMF‐inoculated roots compared to non‐inoculated roots, mainly related to an increased activation of catalases and ascorbate peroxidases. These two enzymes convert the toxic H_2_O_2_ into water molecules, helping ROS scavenging and detoxification (Mhamdi et al. 2010; Pandey et al. 2017). The role of mycorrhizal fungi in ameliorating the root H_2_O_2_ levels has been thoroughly described under both drought and salt stress (Wu and Zou 2009; Estrada et al. 2013; Talaat and Shawky 2014; Huang et al. 2017; Marqués‐Gálvez et al. 2019) and the transcriptional profile of catalases and ascorbate peroxidases is in line with this beneficial effect. Previous reports on tomato have shown the AMF‐mediated induction of catalase, ascorbate peroxidase, peroxidase and superoxide dismutase activities under salt stress (Hajiboland et al. 2010). However, here we show that only catalases and ascorbate peroxidases are upregulated, at least at the transcriptional level. Nonetheless, our analyses focused on overall trends, and the role of specific genes, such as the catalase isozyme encoding gene *LOC104647214*, the peroxidase encoding gene *LOC101247989*, or the ascorbate peroxidase *APX1* gene, which were among the most differentially regulated genes between non‐inoculated and AMF‐inoculated roots, should be further studied.

Overall, our results show that *F. mosseae* inoculation modulates key transcriptional responses in tomato roots under salt stress, contributing to enhanced ion homeostasis and stress tolerance. Improved Na^+^ exclusion and K^+^ retention in AMF‐inoculated plants are supported by the differential expression of genes involved in Ca^2+^ signalling, the SOS pathway, and K^+^/Na^+^ transport, in line with the improved Na^+^/K^+^ ratios observed. The increased regulation of calmodulins and inositol phosphatases suggests that the mycorrhizal symbiosis may intersect with salt stress signalling pathways, likely through a shared Ca^2+^‐dependent regulatory mechanism. Moreover, the selective upregulation of catalases and ascorbate peroxidases in AM roots indicates a targeted antioxidant response that may contribute to ROS detoxification under saline conditions. While these transcriptional profiles support the role of AMF in priming tomato roots for improved salt stress tolerance, future works are required to explore the functional roles, localisation, and regulatory mechanisms of the identified candidate genes. Ultimately, the identification of these transcriptionally relevant genes, including *SlMOCA1*, *SlNHX2‐4* or *SlHKT1*, among others, offers potential targets for breeding or gene‐editing strategies to support the cultivation of *S. lycopersicum* in salt‐stressed environments.

## Author Contributions

J.E.M.‐G. analyzed and discussed the data, drafted the first version of the manuscript and revised subsequent versions. L.G. conducted the experiments, analyzed the data and drafted the first version of the draft. P.B. conducted the wet lab experiments and drafted the first version of the draft. F.S. analyzed the data and revised a final version of the draft. E.Z. contributed to the experiments and revised a final version of the draft; C.P. conducted the measurements and revised a final version of the draft. S.D.R. harvested the samples for the further analyses. W.C. conducted the measurements and revised a final version of the draft. F.V. contributed to the data analyses and revised a final version of the draft. R.B. conceived and designed the study, contributed to the experiments and revised all versions of the draft.

## Conflicts of Interest

The authors declare no conflicts of interest.

## Supporting information


**Figure S1:** Phenotype of tomato plants at the sampling time point.
**Figure S2:** Relationship between RT‐qPCR and RNA‐seq data.
**Figure S3:** RNA‐seq results of tomato roots in non‐mycorrhizal non‐stressed (NMYC_NS), salt‐stressed (NMYC_SS) and mycorrhizal salt‐stressed (MYC_SS) conditions.
**Figure S4:** Volcano plot of differentially expressed genes from non‐inoculated and AMF‐inoculated tomato roots under salt stress.
**Figure S5:** Expression profile of AMF‐induced genes related to the Common Symbiosis Pathway (CSP).
**Figure S6:**. Unique and overlapping differentially expressed signal transduction annotated genes from non‐inoculated and AMF‐inoculated roots under salt stress.

**Table S1:** Primers used in this study for RT‐qPCR analysis of selected genes.
**Table S2:** Expression profile of the selected housekeeping genes.
**Table S3:** Stress‐related metabolite content in *Solanum lycopersicum* leaves under different treatments.
**Table S4:** Fold change with SE of each gene in each treatment in NS and SS conditions.
**Table S5:** Summary statistics of RNA‐Seq alignment of tomato reads.
**Table S6:** Cluster membership of tomato genes according to their expression pattern in response to arbuscular mycorrhizal colonisation and salt stress.
**Table S7:** Raw RNA‐seq reads count.
**Table S8:** Significant (Padj < 0.05) DEGs in NMYC_SS.
**Table S9:** Significant (Padj < 0.05) DEGs in MYC_SS.
**Table S10:** Significant DEGs (Padj < 0.05) up‐regulated exclusively in not‐mycorrhizal plants under salt stress (NMYC_SS).
**Table S11:** Significant DEGs (Padj < 0.05) up‐regulated exclusively in mycorrhizal plants under salt stress (MYC_SS).
**Table S12:** Significant DEGs (Padj < 0.05) down‐regulated exclusively in mycorrhizal plants under salt stress (NMYC_SS).
**Table S13:** Significant DEGs (Padj < 0.05) down‐regulated exclusively in mycorrhizal plants under salt stress (MYC_SS).
**Table S14:** Significant (Padj < 0.05) DEGs in NMYC_SS and MYC_SS with signal transduction functions.
**Table S15:** Effect of salt stress on ion concentration of potted tomato plants ( *Solanum lycopersicum* cv. Moneymaker), either non‐inoculated or inoculated with the commercial inoculum MycAgro (*Funneliformis mosseae*).
**Table S16:** Significant (Padj < 0.05) DEGs in NMYC_SS and MYC_SS with antioxidant functions.

## Data Availability

The data underlying this article are available in the article and in its Supporting Information published online. RNA‐Seq datasets can be accessed from the NCBI‐SRA repository (accession no. PRJNA1258760).
