# Supplementary material for: Arbuscular Mycorrhiza Induce the Regulation of Ca2+, ROS, and SOS Pathways Under Salt Stress in Tomato Roots
Source: Physiol Plant. 2025 Oct 26;177(6):e70610. doi: 10.1111/ppl.70610 (PMC12554847; doi:10.1111/ppl.70610)
Supplement: Supplementary file 1 — Figure S1: Phenotype of tomato plants at the sampling time point. Figure S2: Relationship between RT‐qPCR and RNA‐seq data. Figure S3: RNA‐seq results of tomato roots in non‐mycorrhizal non‐stressed (NMYC_NS), salt‐stressed (NMYC_SS) and mycorrhizal salt‐stressed (MYC_SS) conditions. Figure S4: Volcano plot of differentially expressed genes from non‐inoculated and AMF‐inoculated tomato roots under salt stress. Figure S5: Expression profile of AMF‐induced genes related to the Common Symbiosis Pathway (CSP). Figure S6:. Unique and overlapping differentially expressed signal transduction annotated genes from non‐inoculated and AMF‐inoculated roots under salt stress. [file PPL-177-e70610-s001.pdf]

Arbuscular mycorrhiza induce the regulation of Ca^{2+}, ROS, and SOS pathways under salt stress in tomato roots.

José Eduardo Marqués-Gálvez[1^*], Luca Giovannini[2^], Pierpaolo Del Boccio[2^], Fabiano Sillo[2], Elisa Zampieri[2], Chiara Pagliarani[2], Walter Chitarra[3], Silvia De Rose[1], Federico Vita[4], Raffaella Balestrini[1*]

[1]Institute of Bioscience and Bioresources, National Council of Research, Via Amendola 165/A, Bari 70056, Italy

[2]Institute for Sustainable Plant Protection, National Research Council (CNR-IPSP), Strada delle Cacce 73, Torino 10135, Italy

[3]Research Centre for Viticulture and Enology—Council for Agricultural Research and Economics (CREA-VE), Via XXVIII Aprile 26, Conegliano (TV) 31015, Italy

[4]University of Bari 'Aldo Moro', Department of Biology, Via Orabona 4, Bari 70124, Italy

^These authors have equally contributed to this work.

*Corresponding author

José Eduardo Marqués-Gálvez

Raffaella Balestrini

E-mail:

joseeduardomarquesgalvez@cnr.it

raffaellamaria.balestrini@cnr.it

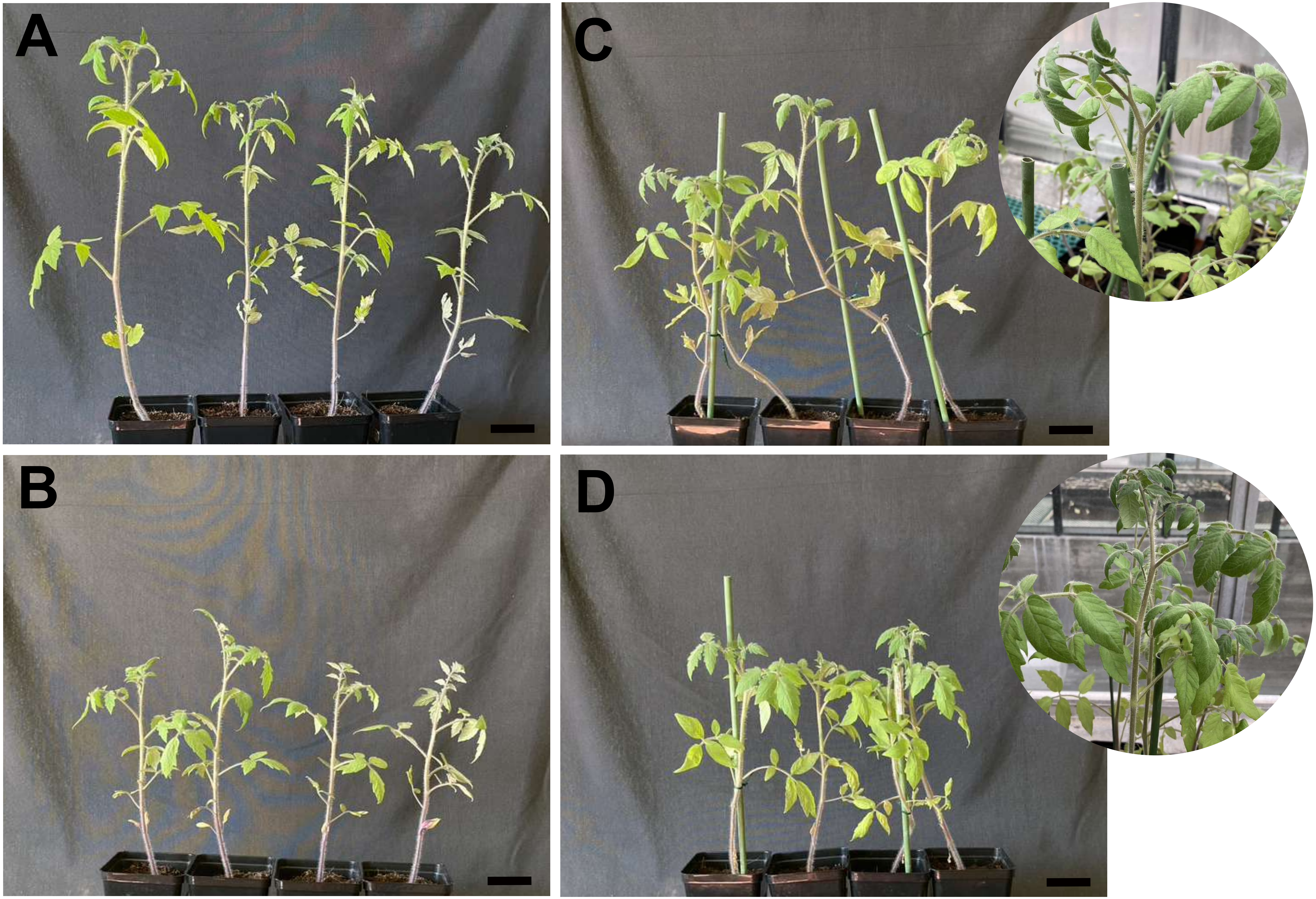
A
B
C
D

Supplementary Figure 1. Phenotype of tomato plants at the sampling time point. (A) Non-inoculated non-stressed plants (NMYC_NS); (B) AMF-inoculated non-stressed plants (MYC_NS); (C) Non-inoculated salt-stressed plants; (D) AMF-inoculated salt-stressed plants. Details of leaves are zoomed in circles for salt-stressed treatments.

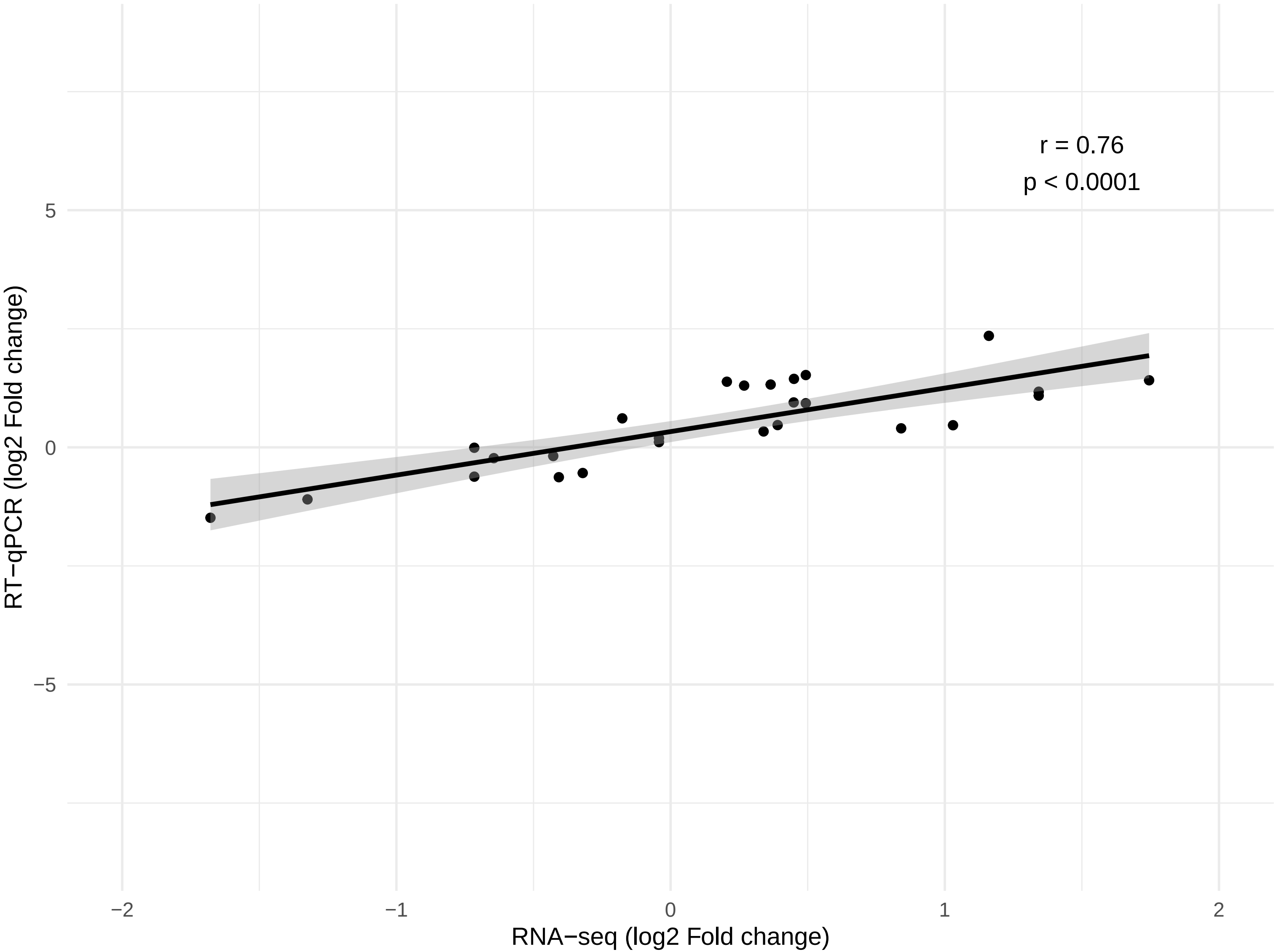
r = 0.76
p < 0.0001
5
0
−5
−2
−1
0
1
2
RT−qPCR (log2 Fold change)
RNA−seq (log2 Fold change)

Supplementary Figure 2. Relationship between RT-qPCR and RNA-seq data. Pearson's correlation between log2 Fold Change (Log2FC) values of RT-qPCR and RNA-seq from the selected Salt Overly Sensitive (SOS), K^+, Na^+, and water transporters and antioxidant genes.

A

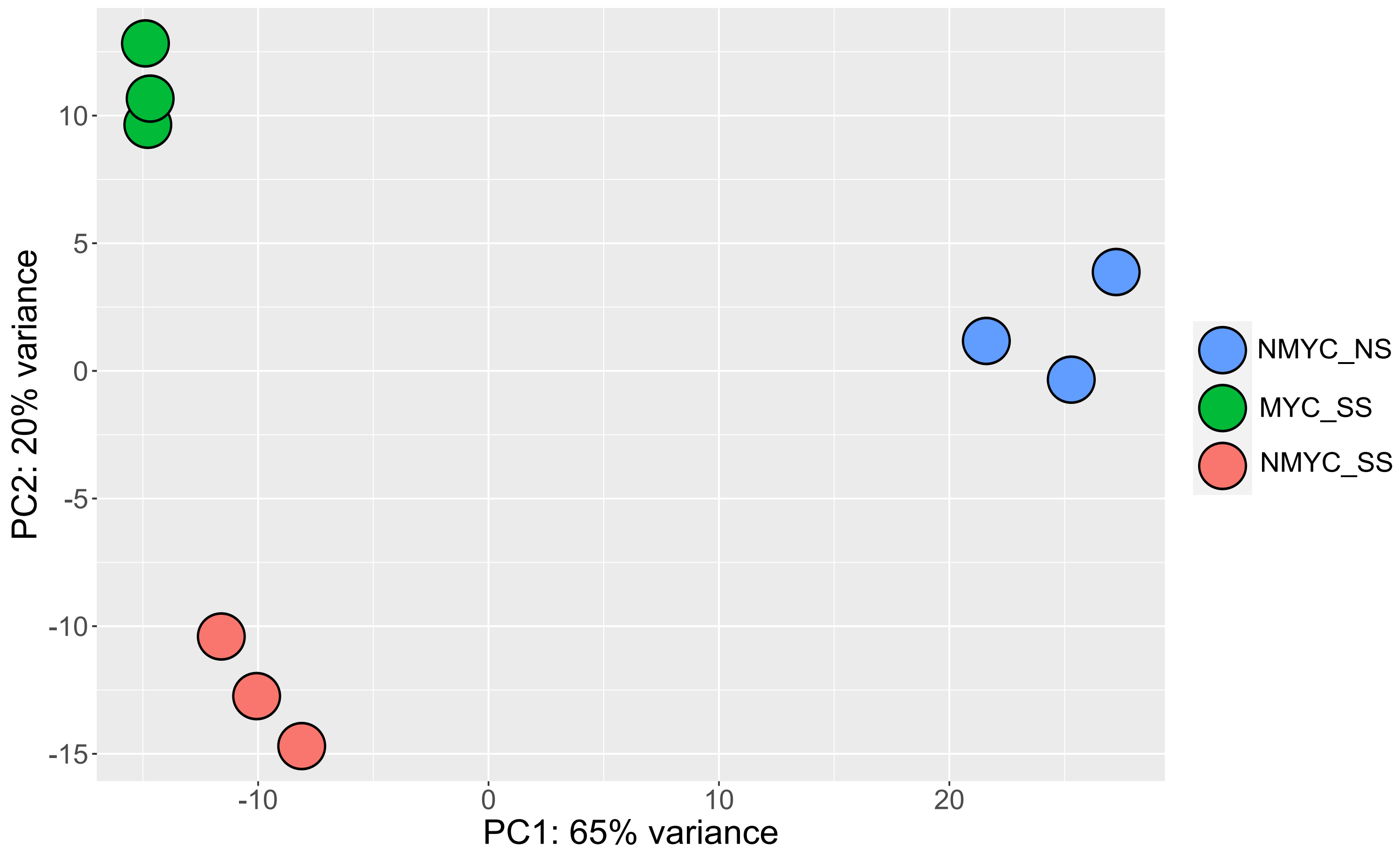

PC2: 20% variance
10
5
0
-5
-10
-15
-10
0
10
20
PC1: 65% variance
NMYC_NS
MYC_SS
NMYC_SS

B

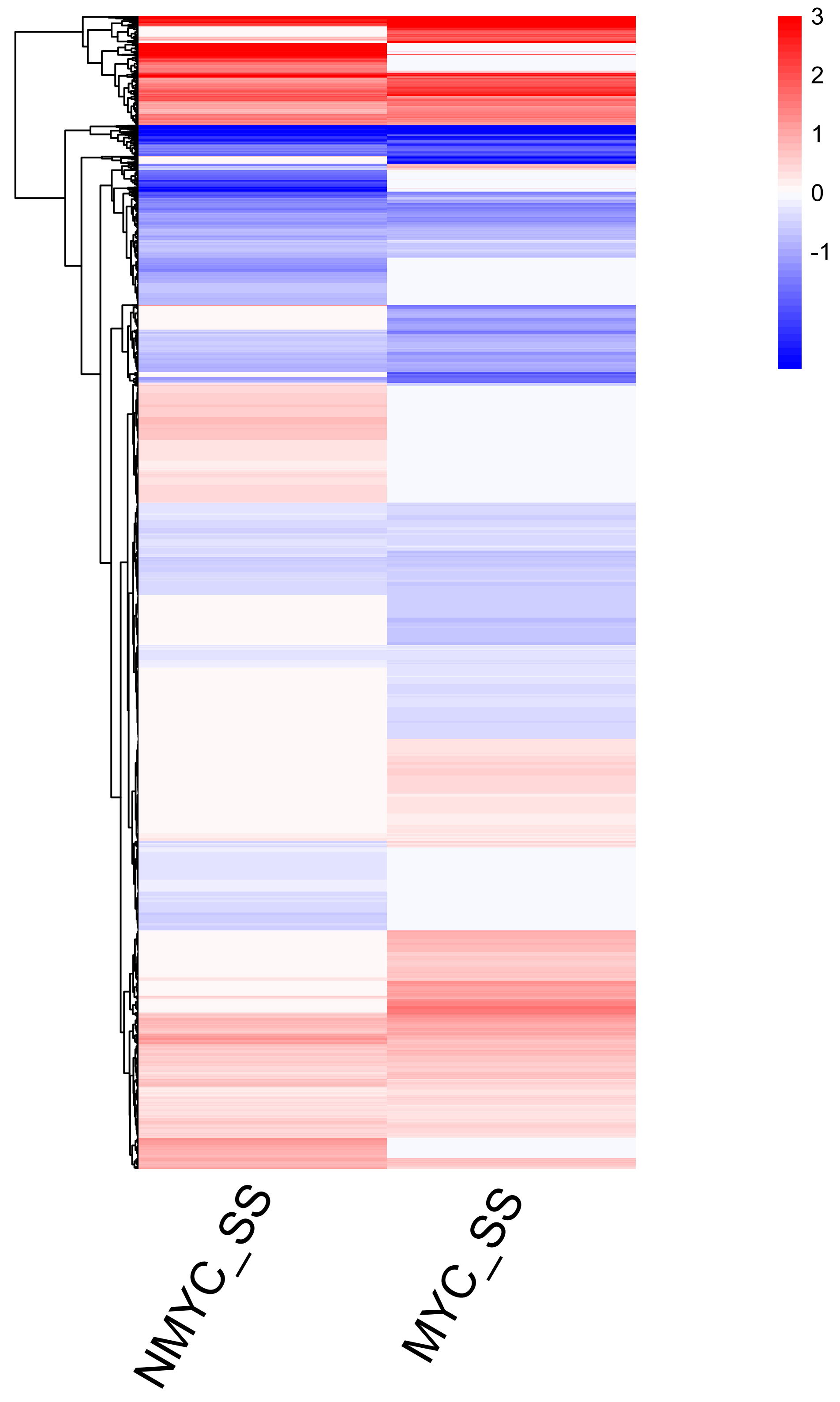

3
2
1
0
-1
NMYC_SS
MYC_SS

Supplementary Figure 3. RNA-seq results of tomato roots in non-mycorrhizal non-stressed (NMYC_NS), salt-stressed (NMYC_SS) and mycorrhizal salt-stressed (MYC_SS) conditions. (A) Principal component analysis (PCA) of each biological replicate included in the experiment according to the expression profile of all *Solanum lycopersicum* genes. Different colours indicate different treatments. (B) Heatmap depicting the normalized expression patterns (Z-scores of Log2FC values) for all genes submitted to DESeq2 analysis. Each row represents a gene, and each column represents a condition. Log2FC values were standardized across each gene (row) to generate Z-scores, highlighting relative up or downregulation across conditions. Red tones indicate higher relative expression (positive Z-scores), and blue tones indicate lower relative expression (negative Z-scores).

A

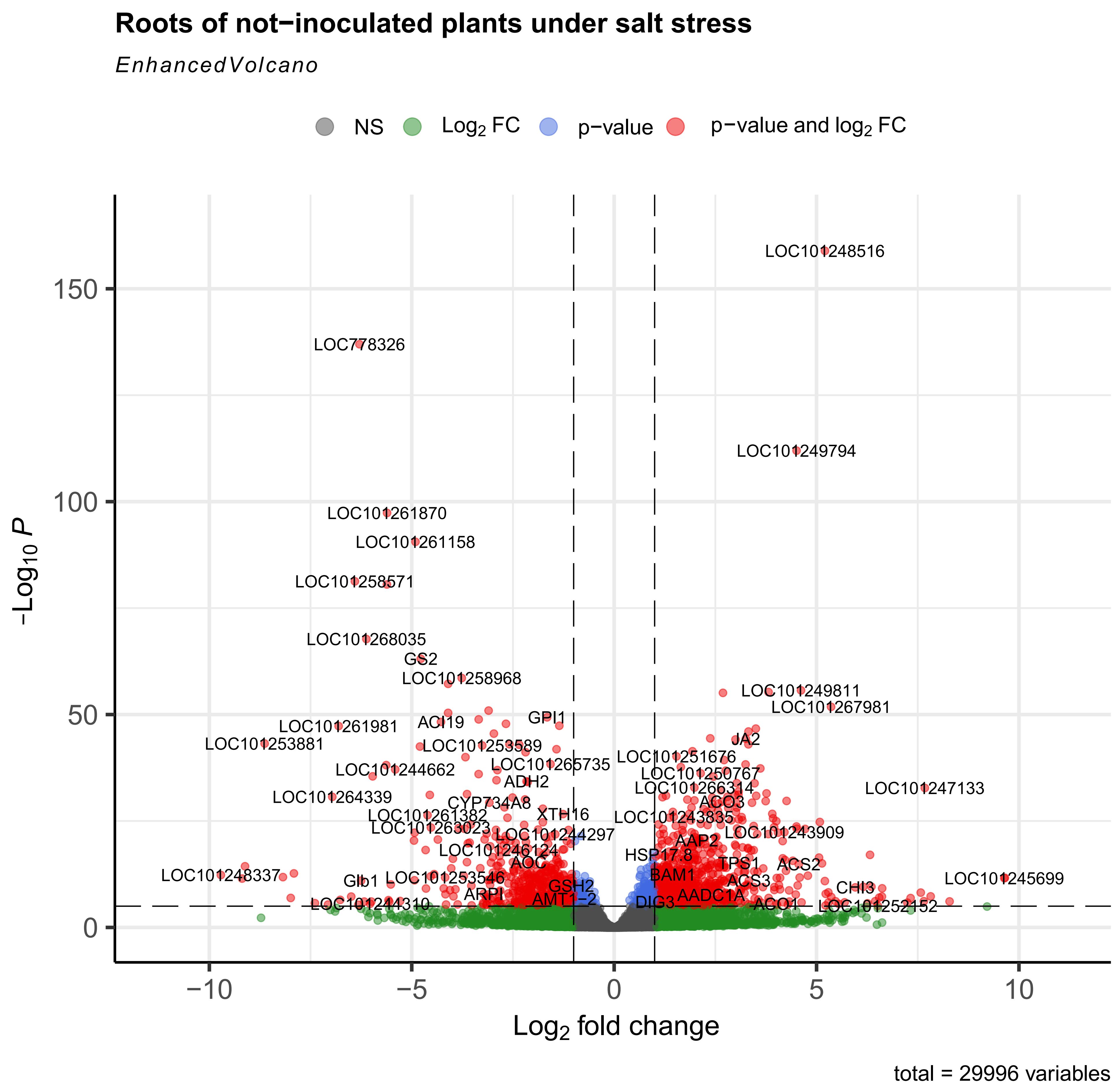
Roots of not−inoculated plants under salt stress
EnhancedVolcano
NS
Log2 FC
p−value
p−value and log2 FC
−Log10 P
Log2 fold change
150
100
50
0
−10
−5
0
5
10
LOC101248516
LOC778326
LOC101249794
LOC101261870
LOC101261158
LOC101258571
LOC101268035
GS2
LOC101258968
LOC101249811
LOC101267981
LOC101261981
ACI19
GPI1
LOC101253881
LOC101253589
JA2
LOC101244662
LOC101265735
LOC101251676
LOC101250767
ADH2
LOC101266314
LOC101247133
LOC101264339
CYP734A8
ACO3
LOC101261382
XTH16
LOC101243835
LOC101263023
LOC101244297
LOC101243909
AAP2
LOC101246124
HSP17.8
AOC
TPS1
ACS2
LOC101248337
Glb1
LOC101253546
BAM1
ACS3
LOC101245699
GSH2
CHI3
ARPI
AADC1A
LOC101244310
AMT1−2
DIG3
ACO4
LOC101252152
total = 29996 variables

B

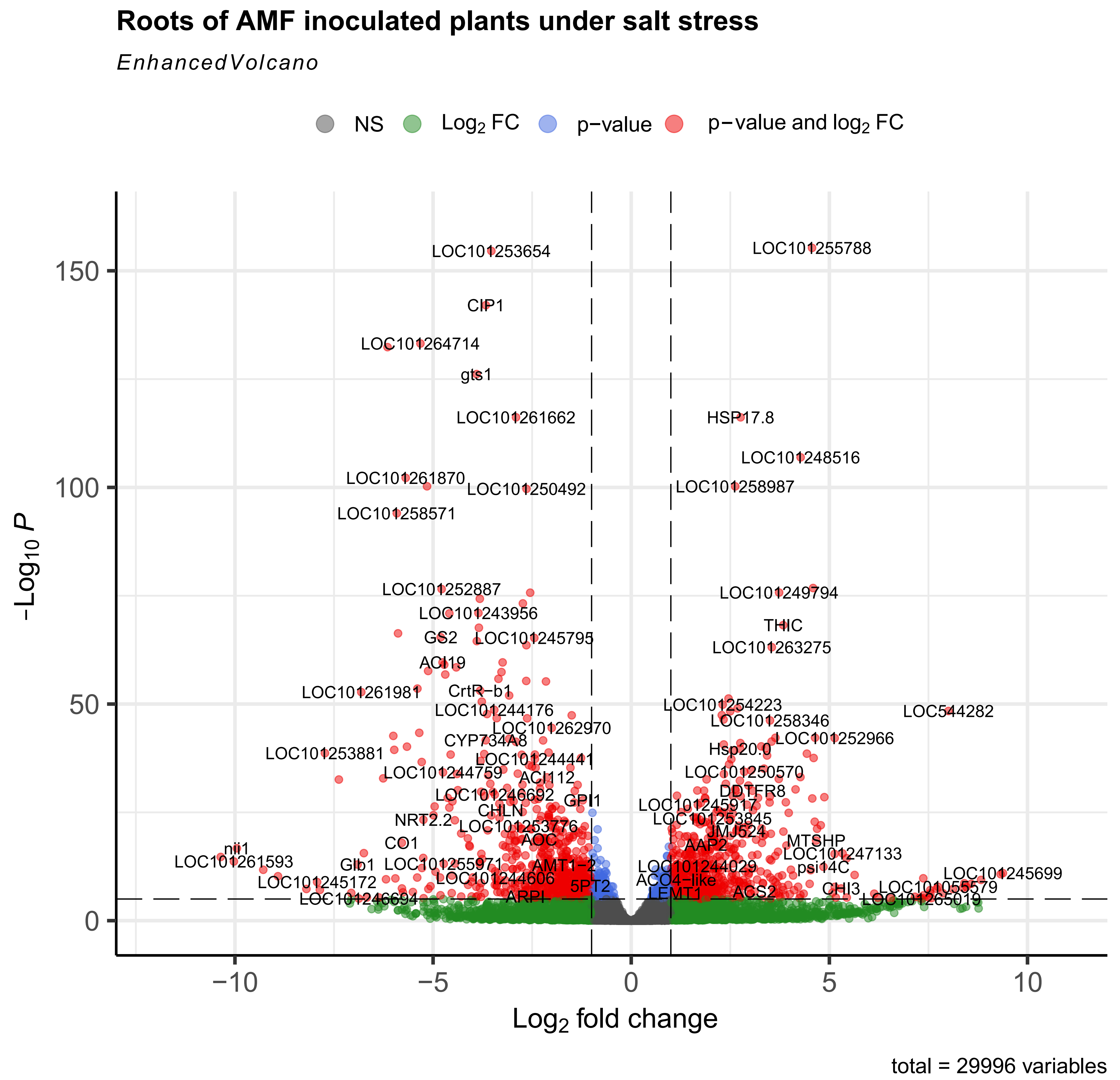
Roots of AMF inoculated plants under salt stress
EnhancedVolcano
NS
Log2 FC
p−value
p−value and log2 FC
−Log10 P
Log2 fold change
150
100
50
0
−10
−5
0
5
10
LOC101253654
LOC101255788
CIP1
LOC101264714
gts1
LOC101261662
HSP17.8
LOC101248516
LOC101261870
LOC101250492
LOC101258987
LOC101258571
LOC101252887
LOC101249794
LOC101243956
THIC
GS2
LOC101245795
LOC101263275
ACI19
LOC101261981
CrtR−b1
LOC101244176
LOC101254223
LOC544282
LOC101258346
LOC101262970
CYP734A8
LOC101252966
LOC101253881
Hsp20.0
LOC101244441
LOC101244759
ACI112
LOC101250570
LOC101246692
DDTFR8
GPI1
CHLN
LOC101245917
NRT2.2
LOC101253776
LOC101253845
CO1
AOC
JMJ524
AAP2
MTSHP
nii1
LOC101247133
LOC101261593
Glb1
LOC101255971
AMT1−2
LOC101244029
psi14C
LOC101245699
LOC101245172
LOC101244606
ACO4−like
CHI3
LOC101055579
SPT2
ACS2
LOC101246694
ARPI
LEMT1
LOC101265019
total = 29996 variables

Supplementary Figure 4. Volcano plot of differentially expressed genes from non-inoculated and AMF-inoculated tomato roots under salt stress. The x axis represents the Log2FC and the y axis the significance level (-log10 p value). Those genes with a significance level higher that 0.05 and a Log2FC higher that 1 are represented in red colour.

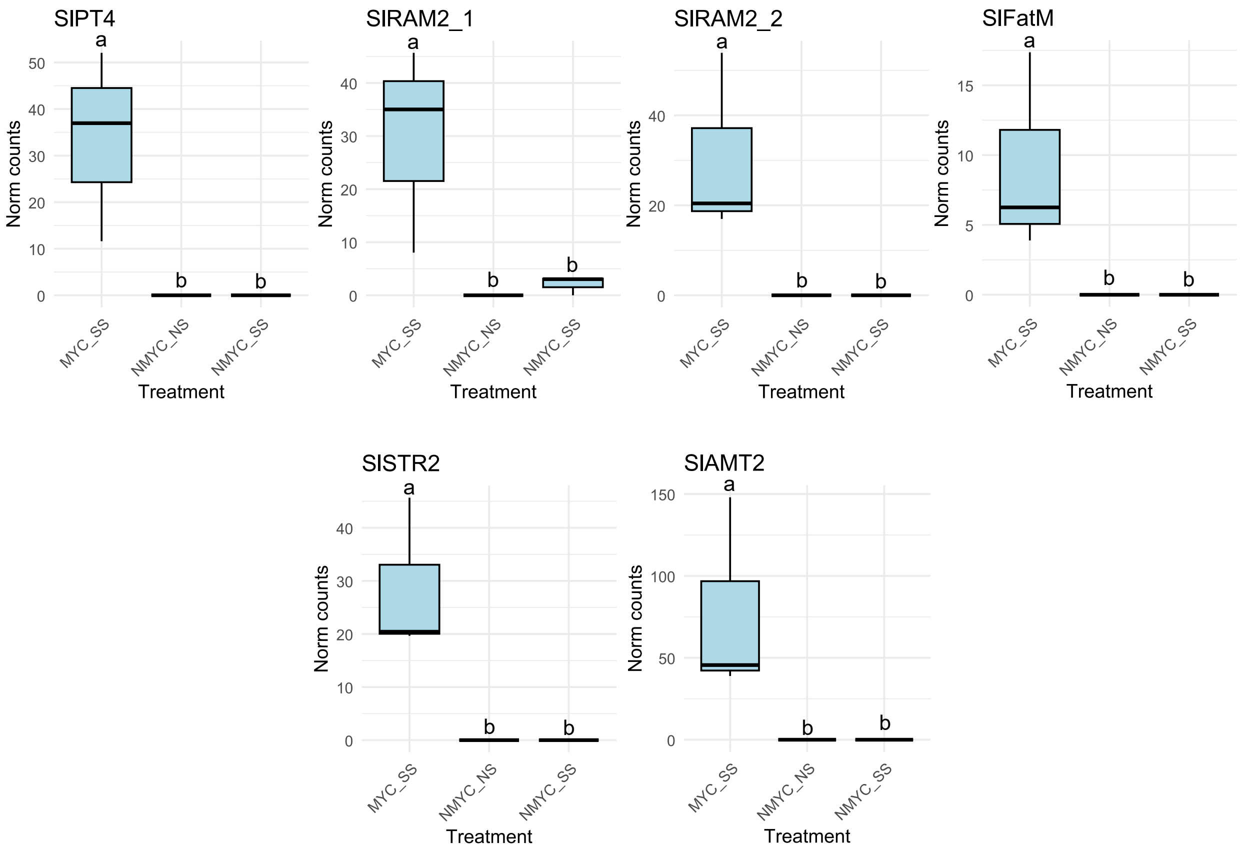
SlPT4
SlRAM2_1
SlRAM2_2
SlFatM
SlSTR2
SlAMT2
Norm counts
Treatment
MYC_SS
NMYC_NS
NMYC_SS
a
b

Supplementary Figure 5. Expression profile of AMF induced genes related with the Common Symbiosis Pathway (CSP). Boxplot represents the DESeq2 normalized counts of *SlPT4*, *SlRAM2*, *SlFatM*, *SlSTR2* and *SlAMT2*. Whiskers represent the limits of the 1.5 interquartile range. Different letters represent significant differences according to their *p*-value based on DEseq2 analysis.

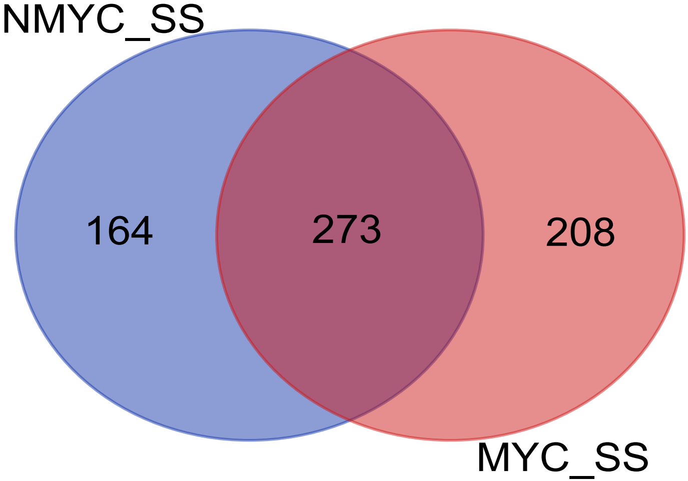
NMYC_SS
164
273
208
MYC_SS

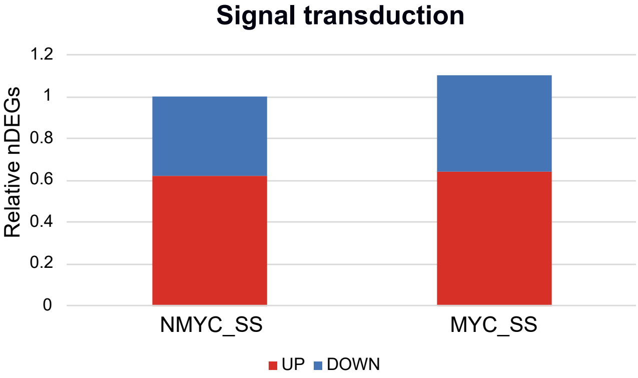
Signal transduction
Relative nDEGs
1.2
1
0.8
0.6
0.4
0.2
0
NMYC_SS
MYC_SS
UP
DOWN

Supplementary Figure 6. Unique and overlapping differentially expressed signal transduction annotated genes from non-inoculated and AMF-inoculated roots under salt stress. At the top, Venn's diagram. At the bottom, barplot indicating the relative number of differentially expressed genes (DEGs).
